# Chromatin insulation orchestrates matrix metalloproteinase gene cluster expression reprogramming in aggressive breast cancer tumors

**DOI:** 10.1186/s12943-023-01906-8

**Published:** 2023-11-28

**Authors:** Pere Llinàs-Arias, Miquel Ensenyat-Mendez, Sandra Íñiguez-Muñoz, Javier I. J. Orozco, Betsy Valdez, Matthew P. Salomon, Chikako Matsuba, Maria Solivellas-Pieras, Andrés F. Bedoya-López, Borja Sesé, Anja Mezger, Mattias Ormestad, Fernando Unzueta, Siri H. Strand, Alexander D. Boiko, E Shelley Hwang, Javier Cortés, Maggie L. DiNome, Manel Esteller, Mathieu Lupien, Diego M. Marzese

**Affiliations:** 1 Cancer Epigenetics Laboratory, Health Research Institute of the Balearic Islands (IdISBa), Palma, 07120 Spain; 2 Saint John’s Cancer Institute, Providence Saint John’s Health Center, Santa Monica, CA USA; 3 Keck School of Medicine, USC Research Center for Liver Diseases, University of Southern California, Los Angeles, CA USA; 4 Science for Life Laboratory, Solna, 17665 Sweden; 5 Advanced Optical Microscopy Facility Scientific and Technological Centres of University of Barcelona, Barcelona, Spain; 6 Department of Pathology, Stanford University School of Medicine, Stanford, CA 94305 USA; 7 Department of Medicine, Cedars-Sinai Medical Center, Samuel Oschin Comprehensive Cancer Institute, Los Angeles, CA 90048 USA; 8 Department of Surgery, Duke University School of Medicine, Durham, NC USA; 9 Pangaea Oncology, Quiron Group, International Breast Cancer Center (IBCC), Barcelona, 08017 Spain; 10 Medica Scientia Innovation Research SL (MEDSIR), Barcelona, 08018 Spain; 11 Department of Medicine, Faculty of Biomedical and Health Sciences, Universidad Europea de Madrid, Madrid, 28670 Spain; 12 Josep Carreras Leukaemia Research Institute, Badalona, Barcelona, Catalonia Spain; 13 Centro de Investigación Biomédica en Red Cancer (CIBERONC), Madrid, 28029 Spain; 14 Institució Catalana de Recerca i Estudis Avançats (ICREA), Barcelona, Catalonia Spain; 15 Physiological Sciences Department, School of Medicine and Health Sciences, University of Barcelona (UB), Barcelona, Catalonia Spain; 16 Princess Margaret Cancer Centre, Toronto, ON M5G 1L7 Canada; 17 Department of Medical Biophysics, University of Toronto, Toronto, ON M5G 1L7 Canada; 18 Ontario Institute for Cancer Research, Toronto, ON M5G 0A3 Canada

**Keywords:** MMP1, MMP8, CTCF, Insulator, Chromatin, Gene regulatory element, cis-regulatory element, Breast cancer, Invasion, ATAC-seq, RNA-seq, Hi-C

## Abstract

**Background:**

Triple-negative breast cancer (TNBC) is an aggressive subtype that exhibits a high incidence of distant metastases and lacks targeted therapeutic options. Here we explored how the epigenome contributes to matrix metalloprotease (MMP) dysregulation impacting tumor invasion, which is the first step of the metastatic process.

**Methods:**

We combined RNA expression and chromatin interaction data to identify insulator elements potentially associated with MMP gene expression and invasion. We employed CRISPR/Cas9 to disrupt the CCCTC-Binding Factor (CTCF) binding site on an insulator element downstream of the MMP8 gene (IE8) in two TNBC cellular models. We characterized these models by combining Hi-C, ATAC-seq, and RNA-seq with functional experiments to determine invasive ability. The potential of our findings to predict the progression of ductal carcinoma in situ (DCIS), was tested in data from clinical specimens.

**Results:**

We explored the clinical relevance of an insulator element located within the Chr11q22.2 locus, downstream of the MMP8 gene (IE8). This regulatory element resulted in a topologically associating domain (TAD) boundary that isolated nine MMP genes into two anti-correlated expression clusters. This expression pattern was associated with worse relapse-free (HR = 1.57 [1.06 − 2.33]; p = 0.023) and overall (HR = 2.65 [1.31 − 5.37], p = 0.005) survival of TNBC patients. After CRISPR/Cas9-mediated disruption of IE8, cancer cells showed a switch in the MMP expression signature, specifically downregulating the pro-invasive MMP1 gene and upregulating the antitumorigenic MMP8 gene, resulting in reduced invasive ability and collagen degradation. We observed that the MMP expression pattern predicts DCIS that eventually progresses into invasive ductal carcinomas (AUC = 0.77, p < 0.01).

**Conclusion:**

Our study demonstrates how the activation of an IE near the MMP8 gene determines the regional transcriptional regulation of MMP genes with opposing functional activity, ultimately influencing the invasive properties of aggressive forms of breast cancer.

**Supplementary Information:**

The online version contains supplementary material available at 10.1186/s12943-023-01906-8.

## Introduction

Breast cancer is the leading cause of cancer death in women [1]. Between 20 and 30% of patients with early breast cancer relapse with distant metastases [2]. However, breast cancer subtypes vary in their aggressivity. For example, triple-negative breast cancer (TNBC) – defined by the lack of estrogen and progesterone receptors as well as the absence of HER2 overexpression or amplification [3] – is associated with worse survival and higher frequencies of lung, brain, and distant nodal relapse compared to other breast cancer subtypes [2]. The ability to invade adjacent tissues and colonize secondary sites through metastasis is associated with at least two-thirds of cancer deaths [4]. The clinical relevance of invasion in breast cancer is not limited to distant metastasis, as the progression from in situ ductal carcinomas (DCIS) [5] to invasive ductal carcinomas (IDC) constitutes an unsolved clinical challenge.

The lack of understanding regarding the molecular determinants involved in the invasion steps often results in overtreatment for patients with early breast cancer or hinders the ability to suppress metastatic progression [6]. Initial invasion, as well as distant metastasis, are tightly regulated by the coordinated activation of gene expression programs. Matrix metalloproteinases (MMPs) are key mediators of invasion [7]. Apart from extracellular matrix (ECM) remodeling, these enzymes promote the release of cytokines or growth factors involved in angiogenesis, epithelial-to-mesenchymal transition (EMT), and inflammation, among others [8]. The human MMP family comprises 23 matrix-degrading enzymes, which are either secreted into the extracellular space or displayed on the cell surface [9]. Control of MMP expression and spatiotemporal distribution is lost during tumorigenesis [10]. The upregulation of a set of MMPs, including MMP1, MMP2, and MMP9, has been associated with worse prognosis in different malignancies including breast cancer [11–13]. Despite the protumorigenic effects of certain MMPs, studies have revealed that other members of the MMP family exhibit different and even opposite roles depending on the context and tumor type. For instance, although MMP8 expression has been linked to poor prognosis in liver and gastric cancers, it surprisingly has a protective effect against metastatic progression in head and neck, skin, and breast cancer [14–16]. These findings challenge the conventional notion that MMPs promote tumor progression and suggest a previously unrecognized protective role [17]. Therefore, gaining more comprehensive knowledge of MMP regulation is crucial to understanding how MMPs contribute to invasion.

MMP expression is altered through epigenetic mechanisms in cancer. At least 14 MMPs show a CpG island in their promoter region, where aberrant DNA methylation is associated with a loss of MMP expression in different cancers [18]. For instance, MMP2 and MMP9 expression levels are restored upon DNA methyltransferase inhibition with 5-aza-2′-deoxycytidine in pancreatic and breast cancer cell lines, respectively [19, 20]. Besides promoter silencing, chromatin conformation may influence transcriptional programs by modulating gene regulatory elements such as insulators and enhancers.

In this study, we have focused on the role of gene regulatory elements in the regulation of MMP expression on a genomic region (Chr11q22.2) that encodes nine MMPs. The combination of multi-omic assays and functional experiments revealed that disruption of an insulator element located near the *MMP8* gene triggers changes in regional gene promoter accessibility, gene expression, and chromatin conformation. Interestingly, the MMP8 insulator element impairment leads to a decrease in the pro-invasive enzyme MMP1 and increased MMP8 expression levels, two events that are associated with antitumor activity in breast cancer. Functionally, we determined that these changes decrease the invasiveness capability in cellular models. We found that tumors can be classified according to the ratio between the expression of pro-invasive and antitumoral MMP genes encoded in the surroundings of the MMP8 insulator element. This signature is significantly associated with disease-free and overall survival in patients with invasive TNBC, and most importantly, is associated with the progression of DCIS to IDC in clinical specimens. Thus, this study unraveled and characterized a regional regulatory role for an insulator element that defines MMP gene expression reprogramming, which may contribute to the invasive ability of aggressive breast cancer.

## Methods

**Data access, collection, and normalization.** The Cancer Genome Atlas (TCGA) data, including mRNA expression and clinical data, were obtained using TCGAbiolinks R package. Datasets were curated to define those patients with TNBC cancer, defined by the negativity of estrogen and progesterone receptors and HER2 [22, 23]. Moreover, only samples with a tumor purity higher than 66% were included. Tumor purity was assessed based on Aran et al. [24]. Chromatin Interaction Analysis with Paired-End Tag (ChIA-PET) data was obtained from Long-range chromatin interaction experiments in public tracks and the WashU Epigenome Browser [25]. Survival analysis was performed using the KM plotter web tool [26]. Overall survival and relapse-free survival were determined using gene chip breast cancer data. For survival analysis, TNBC patients were defined as those classified as ER and HER2 negative in array samples. The follow-up was restricted to 60 months. Kaplan Meier curves were performed on July 6^th,^ 2022. Expression quantitative trait loci (eQTLs) associated with each MMP located at the Chr11q22.2 were downloaded from the Genotype-Tissue Expression (GTEx) Project [27]. Plotted arcs start at the single nucleotide position (SNP) position and end at the TSS of the modulated gene. Association p-value is reflected in arc height, whereas arc sense depends on whether the eQTL is associated with an increase or a decrease in expression. mRNA expression levels of breast cancer cell lines were obtained from the Cancer Cell Line Encyclopedia (CCLE) [28]. Data were downloaded from DepMap Public 22Q2 Primary Files. We used two non-coding mutation databases to interrogate mutations at the Chr11q22.2: the PCAWAG consensus callsets for SNV/Indel [29] (N = 2,658) and whole genome sequencing data of 237 TNBC samples [30]. CTCF binding sites were considered from the intersection between CTCF motifs and the Insulator elements from Figure S3a pipeline. Data processing and visualization were performed using *corrplot*, *ggbio*, *ggpubr*, *patchwork*, *pheatmap*, *rstatix*, *RColorBrewer, tidyverse*, and *viridis* R packages.

**Cell lines.** Cancer cell lines MDA-MB-231 and MDA-MB-436 were purchased from the American Type Culture Collection (ATCC). MDA-MB-231 and MDA-MB-436 were cultured in RPMI 1640 Glutamax™ supplemented with 10% fetal bovine serum (FBS) and 1% penicillin/streptomycin antibiotic at 37 °C and 5% CO_2_. Cell lines were validated with short tandem repeat analysis using the Genetics Core, University of Arizona (Phoenix, USA). All cell lines and models were periodically verified as negative for mycoplasma contamination using MycoAlert Mycoplasma Detection Kit (Lonza).

**In situ and invasive breast cancer clinical tissue gene expression profiling.** The SCAN-B cohort [31] included invasive and in situ BC tissues, that were processed as described [32]. Cases without data about tumor size (T), relapse and/or follow-up under three years, or missing relapse data were removed from the analysis. The relapse-free event was used as an endpoint. Read counts were normalized by applying Log2(Counts + 1). The expression of each MMP gene was standardized using the gene median expression in the entire cohort, and non-detectable levels of any of the MMPs were replaced by their minimal detected signal for each given gene. This cohort contains 85 DCIS, from which 14 progressed into IDC; and it also includes 3620 invasive ductal carcinomas with different tumor sizes (T1 [n = 2,558], T2 [n = 1,006], and T3 [n = 56]). Kaplan-Meier analyses were performed using the DCIS from the same cohort and the receiver operating curves (ROC) were obtained using the pROC v1.16.2 R package. Additionally, the MMP expression was also interrogated in TBCRC 038, a second cohort that patients with DCIS with (n = 121) or without (n = 95) secondary ipsilateral breast events (DCIS or invasive breast cancer) [33]. Data from differentially expressed genes comparing DCIS with or without secondary ipsilateral breast events were used to evaluate MMP expression between these tumor types.

**Additional methods information.** Detailed procedures regarding model generation (both IE8 disruption and hMMP1/MMP1mut ectopic expression), copy number alterations and cell-type enrichment analysis, CUT&RUN, qPCR, multi-omic experiments (Hi-C, ATAC-seq, and RNA-seq), MMP1 and MMP8 protein level determination, and functional assays (including fatty acid uptake, MMP1 activity, cell proliferation, colony formation, wound healing assay, collagen-type I degradation, anchorage-independent growth assay, and collagen-based cell invasion assay) were provided in **extended methods**.

## Results

### MMP dysregulation is associated with relapse-free and overall survival

**Fig. 1:**
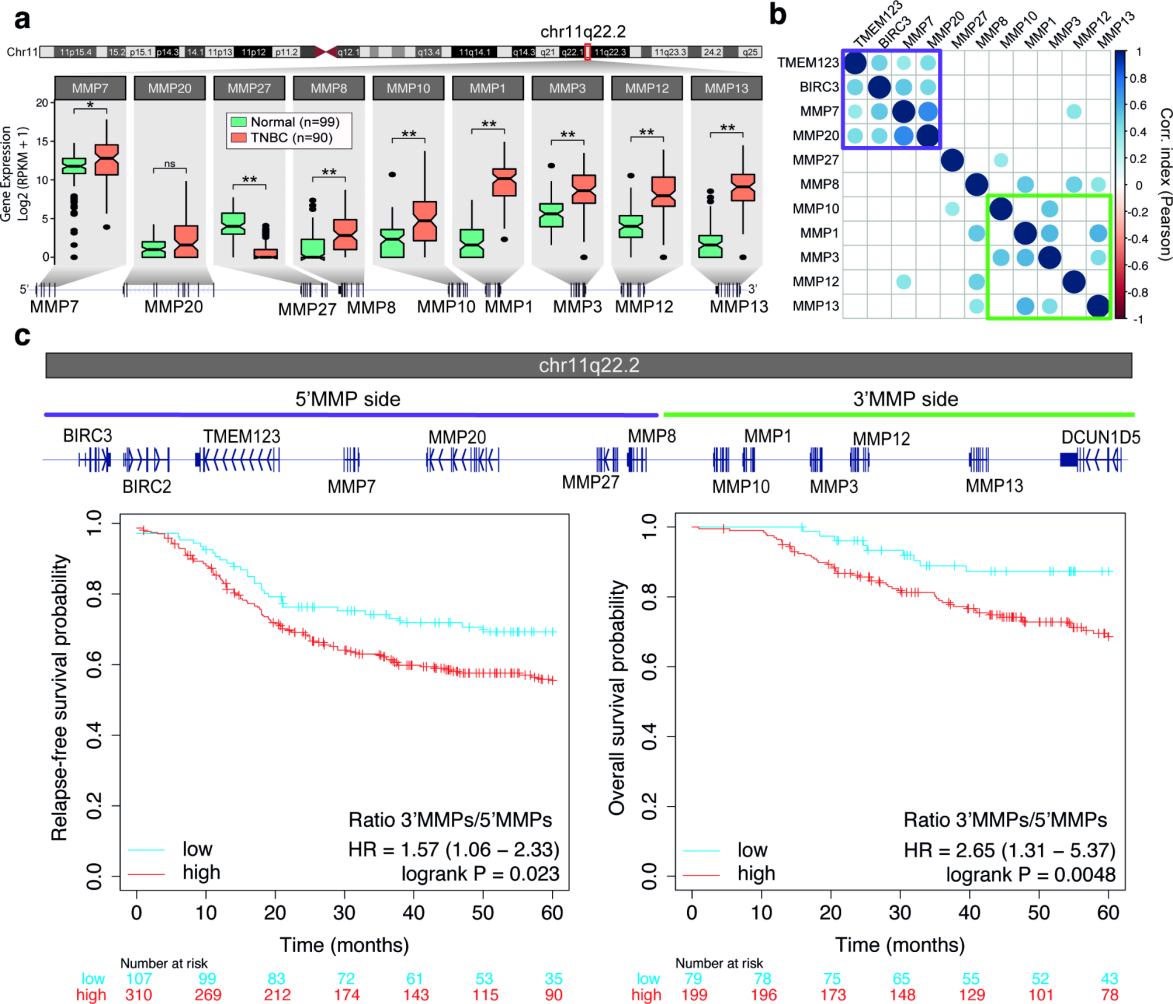
MMP expression and its clinical relevance in TNBC. **a.** Gene expression of MMP genes located on MMP region (chr11q22.2) comparing normal (N = 99) and TNBC (N = 90) patient-derived samples from TCGA consortium. Mann-Whitney test. ns: no significant, *P < 0.05, **P < 0.01. **b.** Correlation matrix for the expression genes located at the MMP locus located genes (P < 0.01). **c.** Gene distribution at MMP region (**top**). Genes were classified according to their location as 5’ or 3’. Gene location was used to define MMP signatures to perform Kaplan-Meier curves of relapse-free survival (**left**) and overall survival (**right**)

Given the relevance of MMPs in the first step of the metastatic process, we explored the differences in mRNA expression levels between non-metastatic TNBC and normal tissue samples in The Cancer Genome Atlas (TCGA) cohort. Seven MMPs were downregulated and ten MMPs were upregulated in TNBC tumors (n = 90) compared to normal tissues (n = 99, Figure S1a). Interestingly, six consecutive MMP genes with increased expression in TNBC tumors are encoded on the same genomic locus (Fig. 1a), in a region that harbors nine different MMP genes, located at Chr11q22.2 (Figure S1b). Furthermore, coexpression patterns were observed in TNBC tumors between genes located on each side of the MMP region, revealing two different expression clusters (Fig. 1b), which were defined as 5’MMP region (containing *MMP7, MMP8, MMP20*, and *MMP27*; in addition to neighbor non-MMP genes *TMEM123* and *BIRC3*) and 3’MMP region (containing *MMP1, MMP3, MMP10, MMP12*, and *MMP13*). The ratio of the expression levels between MMP genes located at Chr11q22.2 (3’MMPs/5’MMPs ratio) was significantly higher in TNBC (Figure S1c).

We wondered whether MMP expression differences might be a consequence of chromosomal or regional copy number alterations (CNAs). Despite TNBC and HER2 + exhibiting significant changes in copy number compared to the HR + subtype (Figure S2a), this alteration was not correlated with the expression of genes located at the MMP locus (Figure S2b). We also explored the potential influence of tumor heterogeneity on the MMP expression signatures. Using transcriptome deconvolution to estimate the cellular composition of each tumor tissue [34], we found that none of the MMP signatures displayed a correlation with stromal content. 5’MMPs and 3’MMPs signatures showed a poor correlation with immune infiltration (r = 0.23 and 0.27, respectively) and TME content (r = 0.26 and 0.33, respectively). Importantly, the 3’MMPs/5’MMPs ratio signature was independent of stroma content, immune cell score, and microenvironment content (Figure S2c).

Thus, we hypothesized that the alternative expression of these two clusters might be associated with clinical outcomes. In the survival analyses of patients with TNBC (n = 417), we observed that tumors with a higher 3’MMP/5’MMP ratio have a significantly shorter relapse-free survival (RFS; Fig. 1c, [log rank P = 0.023; hazard ratio (HR) = 1.57, 95% CI = 1.06 − 2.33]) and overall survival (OS, Fig. 1c, [log rank P = 0.005; HR = 2.65, 95% CI = 1.31 − 5.37]). We also conducted survival analyses considering the expression of 5’MMPs alone or 3’MMPs alone instead of the ratio. We observed that while the expression of 5’MMP genes was associated with better RFS and OS, the expression of 3’MMP genes did not show an association with survival (Figure S3a). Importantly, the evaluation of patients affected by other breast cancer subtypes showed that the 3’MMPs/5’MMPs ratio was also associated with a worse prognosis in hormone receptor-positive breast cancer, but not in patients with HER2-positive disease. Interestingly, when considering non-breast solid tumors, this signature was associated with a worse prognosis in liver cancer, lung adenocarcinoma, and sarcoma, whereas it correlated with better survival in gastric cancer (Figure S3b).

### An insulator element near the MMP8 gene promoter region is involved in regional MMP gene regulation

**Fig. 2:**
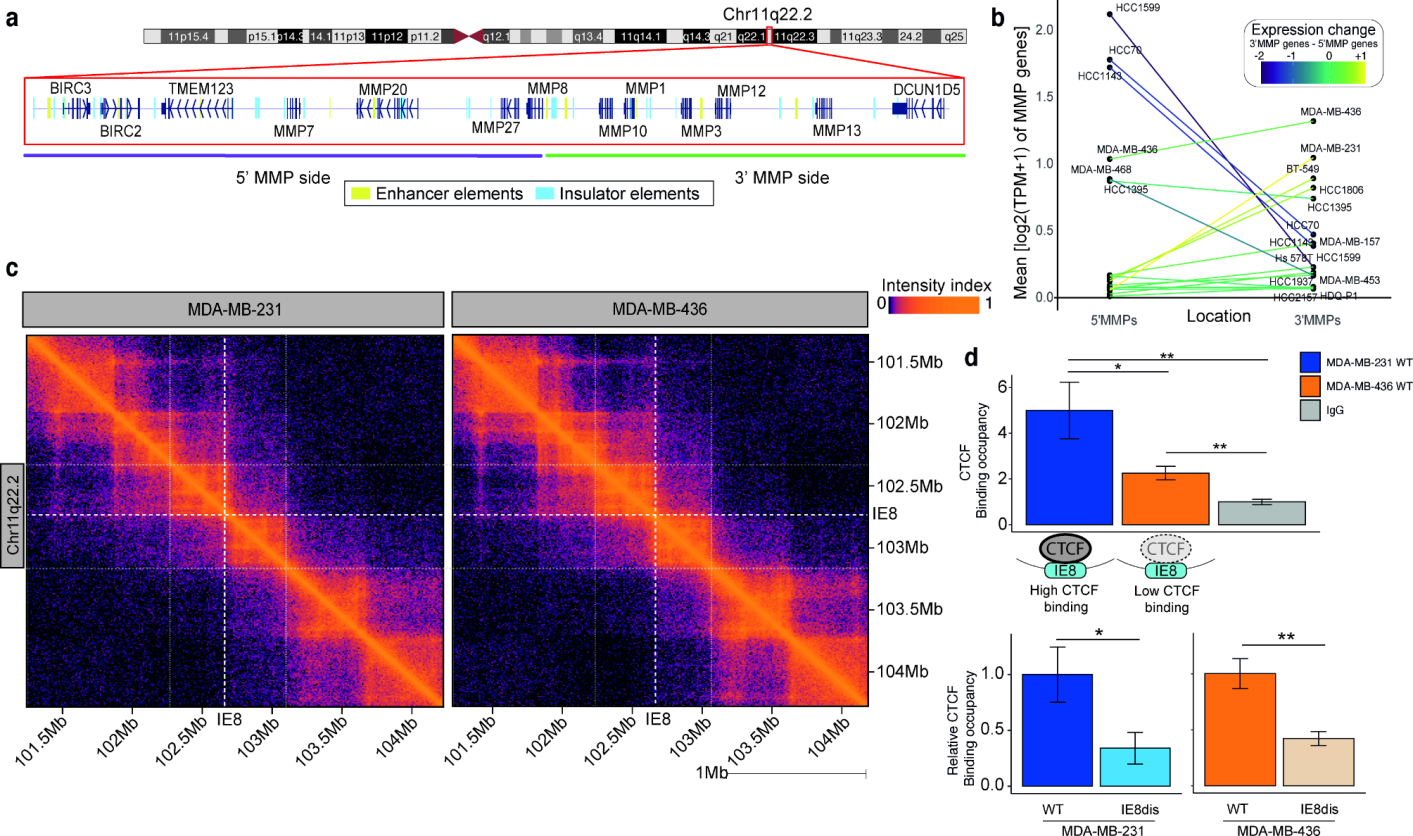
Identification and edition of gene regulatory elements at chr11q22.2. **a.** Scheme of identified enhancer and insulator elements. CRISPR/Cas9-disrupted Insulator element 8 (IE8) is highlighted. **b.** Expression profile of 5’ and 3‘MMP genes in TNBC cell lines. **c.** Hi-C contact frequency matrix for the 3 Mb genomic region surrounding IE8 binned at 10-kb resolution. **d.** CTCF binding is represented as fold enrichment (**top**) relative to isotype control in MDA-MB-231 and MDA-MB-436 and (**bottom**) relative fold enrichment after IE8 disruption. Student’s T-test. *P < 0.05, **P < 0.01

Considering the expression pattern exhibited by the MMP genes at Chr11q22.2, we explored the distribution of gene regulatory elements in this region (Figure S4a). We found 29 potential insulator elements (IEs) and 13 potential enhancer elements (EEs) at Chr11q22.2 (Fig. 2a). The IEs play a pivotal role in the topologically associating domain (TAD) formation, being DNA elements recognized by the CCCTC-binding factor (CTCF) and contributing to chromatin loop formation [35]. We explored potential TADs at Chr11q22.2 using data from CTCF ChIA-PET (Figure S4b). This data suggested that the IE located between the *MMP8* promoter region and the *MMP10* gene body (Chr11:102,732,800 − 102,733,900; hg38), hereinafter IE8, demarcates a boundary for a TAD contributing to the expression signature observed by MMP genes at Chr11q22.2. This is supported by data from eQTLs since we observed that SNPs tend to modulate the expression of genes located on the same side of TADs at Chr11q22.2 (Figure S4c). It is worth noting that the CTCF binding site of the IE8 was depleted in mutations according to data from the Pan-Cancer Analysis of Whole Genomes (PCAWAG) database (N = 2,658) and whole genome sequencing of TNBC samples (n = 237) (Figure S4d), indicating that its normal function is required in cancer.

### Construction of cell models to explore the impact of IE8 activation on MMP gene expression

To select cell models that resemble the expression patterns detected in clinical specimens, we classified TNBC patients based on the expression of MMP genes into high 3’MMP/5’MMP ratio, intermediate 3’MMP/5’MMP ratio, and normal-like MMP expression profile (Figure S5a). Following, we profiled the expression levels of MMP genes in a panel of well-characterized TNBC cell lines (n = 17; Fig. 2b) and selected two cell lines that recapitulate the most predominant MMP abnormal expression patterns in TNBC clinical specimens: high 3’MMP/5’MMP ratio (MDA-MB-231) and intermediate 3’MMP/5’MMP ratio (MDA-MB-436). Chromatin interactions were established using the Hi-C method in the two cell models. Interestingly, suggesting an active insulator role, we observed that IE8 colocalizes with TAD boundaries in both models (Fig. 2c), in concordance with data from ChIA-PET.

Both TNBC cell lines were transiently transfected with a Cas9-containing plasmid and a sgRNA to stably disrupt the CTCF binding motif on IE8 using CRISPR/Cas9 technology. A single clone for each condition was selected to perform further experiments (Figure S5b). The CTCF binding ability to IE8 was tested in both cell lines through Cleavage Under Targets and Release Using Nuclease (CUT&RUN) followed by qPCR [36]. Basal levels of CTCF occupancy were higher in MDA-MB-231 than in MDA-MB-436, showing the biological variability reflected in clinical specimens. After IE8 disruption, a significant decrease in CTCF binding was observed in both cell lines (Fig. 2d).

In addition, we performed Hi-C on the cell models after IE8 disruption. We found that disruption of IE8 did not change the higher-order TAD chromosomal organization (Figure S6a). However, the distribution of Hi-C interaction signals indicates that IE8 interacts with 3’MMP distal regulatory regions in both models (Figure S6b). Interestingly, some of these interactions contain additional IEs disposed of in a convergent orientation to IE8, which is a requirement for IE interactions [37]. Thus, we identified that insulation induced by IE8 activation preferably involves IEs located at the 3’MMP region of the MMP locus Chr11q22.2 (Figure S6b). Apart from regional interactions, we also explored high-confidence interchromosomal interactions in both models (Figure S6c). Interestingly, we found interchromosomal interactions between the two ends of the MMP locus and super-enhancer elements located in different chromosomes (Figure S6d).

### Disruption of IE8 leads to local chromatin accessibility changes

**Fig. 3:**
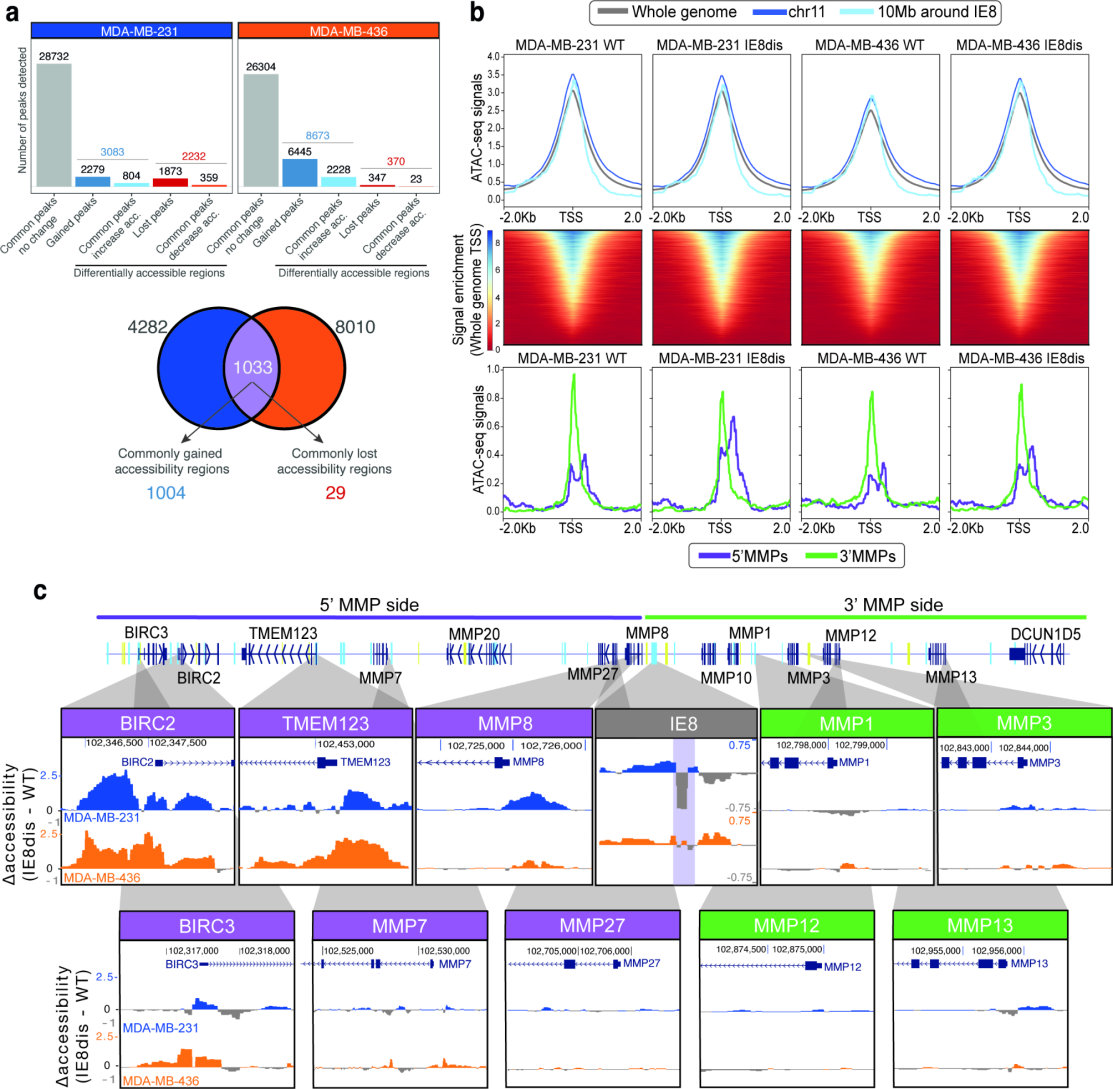
Local consequences of IE8 disruption. **a.** (**top**) Description of differentially accessible regions in MDA-MB-231 and MDA-MB-436 upon IE8 disruption and (**bottom**) representation of commonly modulated regions in both cell lines. **b.** (**top**) ATAC-seq peak intensity signals of all the TSS present in the whole genome, chr11 and 10 MB around IE8 (**middle**) Heatmap of active TSS in TNBC models and (**bottom**) variations on the accessibility of promoter regions of 5’ MMPs (**purple**) and 3’MMPs (**green**) regions in MDA-MB-231 and MDA-MB-436 before and after IE8 disruption. **c.** Illustrative examples of variation in accessibility upon IE8 disruption (IE8dis minus WT) in the IE8 region (**grey**), the 5’ MMP region (**purple**), and the 3’MMP region(**green**)

To address the implications of the IE8 disruption on chromatin accessibility, we performed ATAC-seq on our TNBC cell line models. 34,047 common peaks between all replicates were identified in MDA-MB-231, whereas 35,347 common peaks were found in MDA-MB-436. Accessibility analysis was performed by assessing differential accessibility peaks, which were defined by either their presence in only one condition (WT or IE8 dis) or by the significant change in intensity of shared peaks between conditions (Figure S7a). Thus, we identified 3,083 and 2,232 regions that gained and lost accessibility upon IE8 disruption in MDA-MB-231, respectively. Regarding MDA-MB-436, 8,673 and 370 regions were more and less accessible after IE8 disruption, respectively (Fig. 3a). Importantly, a significant overlap between the differentially accessible regions (n = 1,033) of both cell line models was observed.

Despite changes in chromatin accessibility being detected across the genome, we examined whether they were also specifically enriched around IE8. We explored the differentially accessible regions located on chromosome 11 – where our region of interest is located – as well as in different width windows around IE8 (± 10 MB to ± 0.5 MB). Significant enrichment in the number of differentially accessible regions was observed in the IE8 genome vicinity whereas no differences were observed across chromosome 11 (Figure S7b).

Moreover, we aimed to identify potential gene regulatory elements associated with differential chromatin accessibility reprogramming after IE8 disruption. The relative abundance of promoter, enhancer, and insulator elements that exhibited differential accessibility after IE8 disruption was similar between MDA-MB-231 and MDA-MB-436 (Figure S7c). We focused on changes in chromatin accessibility around (± 2 kb) the gene transcription start sites (TSS). As expected, the resulting heatmap displayed a similar accessibility profile on all conditions with increased peak density on the TSS (Fig. 3b). Similar profiles were also observed between all conditions when peaks were centered in insulators and enhancers (Figure S7d). However, we found interesting differences when we focused on the TSS of the genes located at the Chr11q22.2. Promoters in the 5’MMP region were more accessible upon IE8 disruption, but no changes were observed in promoter regions located toward the 3’MMP region (Fig. 3b and c). In this regard, we also found changes in the accessibility of enhancer elements in both 5’ and 3’ MMP regions (Figure S7e). We additionally identified a decrease in chromatin accessibility at the IE8 CTCF binding site disrupted by CRISPR/Cas9 (Fig. 3c). In agreement with a higher CTCF occupancy detected by CUT&RUN (Fig. 2d), the decrease in chromatin accessibility of this site was more evident in the MDA-MB-231 cells than in the MDA-MB-436 cells.

### IE8 disruption modulates regional MMP expression patterns

**Fig. 4:**
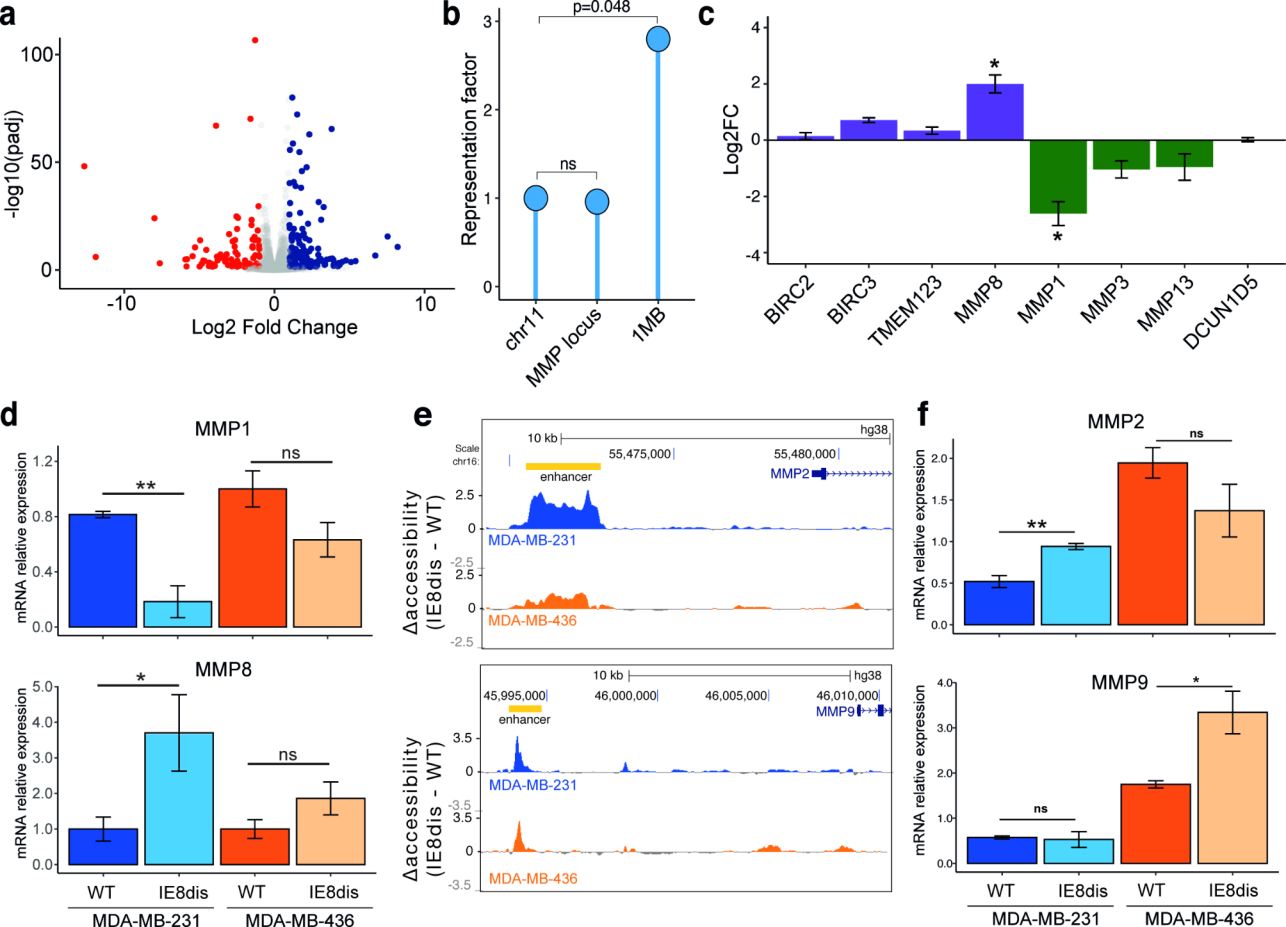
mRNA expression changes after IE8 disruption. **a.** Volcano plot summarizes the RNA-seq results in MDA-MB-231. 166 genes were significantly upregulated (Padj < 0.05) (**blue dots**), whereas only 71 were downregulated (**red dots**). **b.** Representation factor of differentially expressed genes in MDA-MB-231. Hypergeometric test. ns P > 0.05. **c.** Variation of MMP8 and MMP1 RNA expression in MDA-MB-231 in RNA-seq. Student’s T-test. *P < 0.05, **P < 0.01. **d.** MMP1 (**top**) and MMP8 (**bottom**) mRNA expression levels determined by qPCR in MDA-MB-231 and MDA-MB-436 models. Student’s T-test. ns P > 0.05, *P < 0.05, **P < 0.01 **e.** Variation of accessibility in enhancers close to MMP2 (**top**) and MMP9 (**bottom**) promoters. **f**. MMP2 (**top**) and MMP9 (**bottom**) mRNA expression levels determined by qPCR in MDA-MB-231 and MDA-MB-436 models. Student’s T-test. ns P > 0.05, *P < 0.05, **P < 0.01

We determined whether the observed alterations in chromatin accessibility resulted in differential expression of the MMP genes near IE8. We, therefore, assessed the mRNA expression levels of MMP genes in the wild-type and IE8-disrupted cells through RNA-seq. We observed 237 mRNAs with a significant differential expression upon IE8 impairment (Fig. 4a). We performed a gene ontology (GO) analysis to classify the 166 significantly upregulated and 71 downregulated genes by biological process. Importantly, we found significant enrichment of extracellular matrix organization or Ca^2+^-dependent cell-cell adhesion (Figure S8a). In addition, pathways associated with fatty acid import were also upregulated, which has been confirmed as a feature of TNBC tumors with better prognosis [38]. Therefore, we assessed the functional impact of these gene expression changes by evaluating fatty acid uptake ability. Importantly we found that after IE8 disruption, both cell lines increased fatty acid uptake (Figure S8b). Since the CRISPR/Cas9-mediated IE8 disruption occurred in chromosome 11, we assessed whether significant changes were enriched on this chromosome. We did not observe significant variation in gene expression when considering all the genes, but the genes located 1 Mb around IE8 were significantly modulated after IE8 disruption in MDA-MB-231 (Fig. 4b).

Regarding the Chr11q22.2 encoded genes; the modification of regional gene expression patterns supports the promoter accessibility changes (Fig. 3c). RNA-seq revealed two interesting changes at the mRNA level, an increase in MMP8 and a decrease in MMP1 (Fig. 4c) after IE8 disruption. These alterations were confirmed by qPCR (Fig. 4d). MDA-MB-231 showed a shift between MMP1 and MMP8 after the IE8 disruption, exhibiting a decrease in the pro-invasive enzyme MMP1 and an increase in MMP8, associated with antitumor activity. Although variations were not statistically significant, RNA expression levels displayed a similar tendency in MDA-MB-436 (Fig. 4d). Thus, the ratio between MMP1 and MMP8 is decreased after IE8 disruption, resembling the profile observed in healthy breast samples as opposed to tumor samples (Figure S8c). Importantly, changes in MMP expression after the switch after IE8 disruption are not associated with the reprogramming of interchromosomal interactions (Figure S6d).

We further explored whether reprogramming of the MMPs located in the genomic vicinity of IE8 triggered any compensatory mechanism either modulating the expression of tissue inhibitor of metalloproteinases (TIMP) genes, a four-member family that balances the MMP activity or altering the RNA levels of two other relevant metalloproteinases, MMP2 and MMP9. TIMP1, TIMP2, TIMP3, and TIMP4 did not show significant changes at the mRNA level upon IE8 disruption (Figure S8d). IE8 depletion was not associated with changes in chromatin accessibility at MMP2 or MMP9 promoter regions. However, an important increase in accessibility was reported on enhancer elements located 8 kb and 15 kb upstream of TSS of the MMP2 and MMP9 genes, respectively (Fig. 4e). These changes were translated into a cell line-dependent increase in MMP mRNA levels. MMP2 was upregulated only in MDA-MB-231 whereas MMP9 was increased in MDA-MB-436 after IE8-disruption (Fig. 4f).

We considered whether changes in chromatin accessibility and the concomitant modulation of gene expression may also be observed in TNBC patient samples. We explored the six TNBC samples with ATAC-seq and mRNA-expression data available at TCGA. We found different levels of chromatin accessibility at IE8 (Figure S8e). Interestingly, we could correlate these changes to variations in 3’MMP/5’MMP expression. We observed that those patient-derived samples with higher levels of accessibility at IE8 showed a higher ratio of 3’MMPs/5’MMPs (r = 0.87, p = 0.02, Figure S8f).

### IE8 disruption interferes with MMP1 release and decreases invasive potential in Breast cancer

**Fig. 5:**
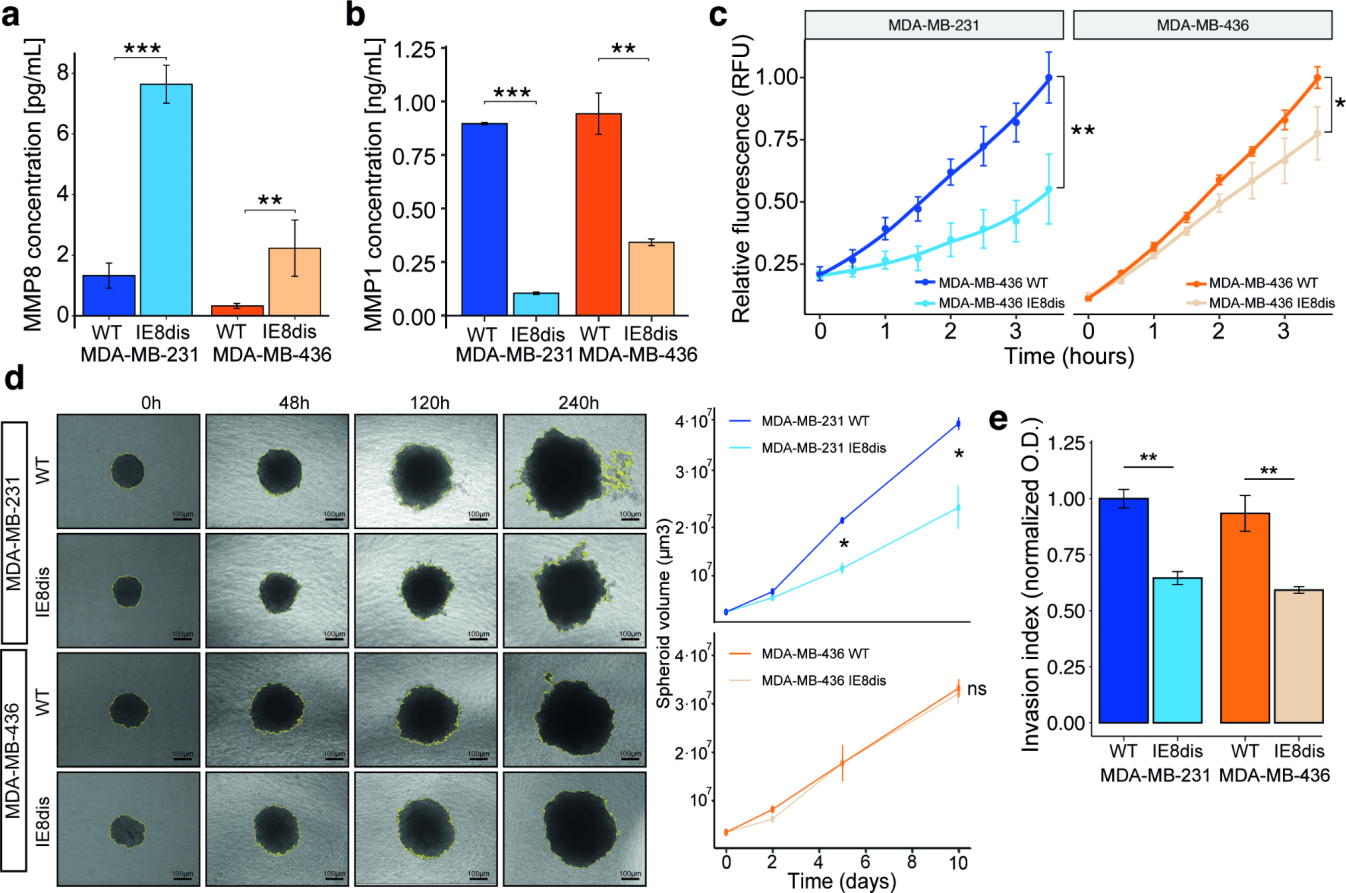
Functional impact of changes in MMP protein expression and activity after IE8 disruption. Evaluation of MMP8 (**a**) and MMP1 (**b**) levels released to the extracellular space after IE8 disruption in MDA-MB-231 and MDA-MB-436. **c.** Time-course of MMP-1 activity in MDA-MB-231 and MDA-MB-436 upon IE8 disruption. **d.** (**left**) An illustrative example of anchorage-independent spheroid growth of MDA-MB-231 and MDA-MB-436 cell models and (**right**) volume quantification. **e**. Colorimetric quantification of cell invasion MDA-MB-231 and MDA-MB-436 cell invasion. Student’s T-test. ns: no significant, *P < 0.05, **P < 0.01, ***P < 0.001. O.D. Optical density

We observed that IE8 disruption triggered a significant increase in MMP8 protein levels both in MDA-MB-231 and MDA-MB-436 (Fig. 5a). Conversely, a decrease in both MMP1 abundance and MMP1 activity was observed upon IE8 disruption (Fig. 5b and c). We checked the functional consequences of IE8 disruption on relevant features of cancer cells. We did not observe differences either in cell proliferation (Figure S9a) or in clonogenic ability (Figure S9b). Migration capacity assessed by wound healing assay showed no changes between wild-type and IE8 disruption conditions (Figure S9c). However, the ability to degrade collagen-type I – which is a major component of ECM and the breast basement membrane [39] – was decreased after IE8 disruption in MDA-MB-231, whereas we did not observe changes in MDA-MB-436 (Figure S9d). Similarly, anchorage-independent growth assay was significantly reduced in IE8-disrupted MDA-MB-231 cells, but no differences were observed in the IE8-disrupted MDA-MB-436 cells (Fig. 5d). In addition, when we employed collagen I, we observed a significant decrease in the number of invasive cells after IE8 disruption in both the MDA-MB-231 and MDA-MB-436 cells (Fig. 5e).

We then evaluated whether the functional consequences changes observed after IE8 are rescued by ectopic expression of MMP1. Thus, MDA-MB-231 and MDA-MB-436 IE8dis cells were transfected with either a functional MMP1 (hMMP1) or an inert catalytic mutant form of MMP1 (MMP1mut, Figure S10a). Ectopic expression of these vectors increased the MMP1 mRNA levels in both cell lines (Figure S10b). As expected, the MMP1 activity was only increased in the cells expressing hMMP1 (Figure S10c). Once the models were validated, we performed functional experiments including cell proliferation, colony formation, migration, and invasion. Ectopic expression of MMP of any of the two variants did not affect cell proliferation (Figure S10d). However, we determined that while hMMP1 increased in the clonogenic ability (Figure S10e) and wound healing rate (Figure S10f), the MMP1mut showed no differences with the IE8-disrupted cells. Beyond that, functional MMP1 overexpression on the IE8-disrupted clones triggered an enhanced invasion rate on collagen I-covered membranes (Figure S10g). Altogether, these results suggest that IE8 disruption diminishes invasiveness potential in the presence of collagen type I fibers by orchestrating a reprogramming of the MMP gene expression pattern.

### MMP shift is associated with progression to invasion in early Breast cancer

**Fig. 6:**
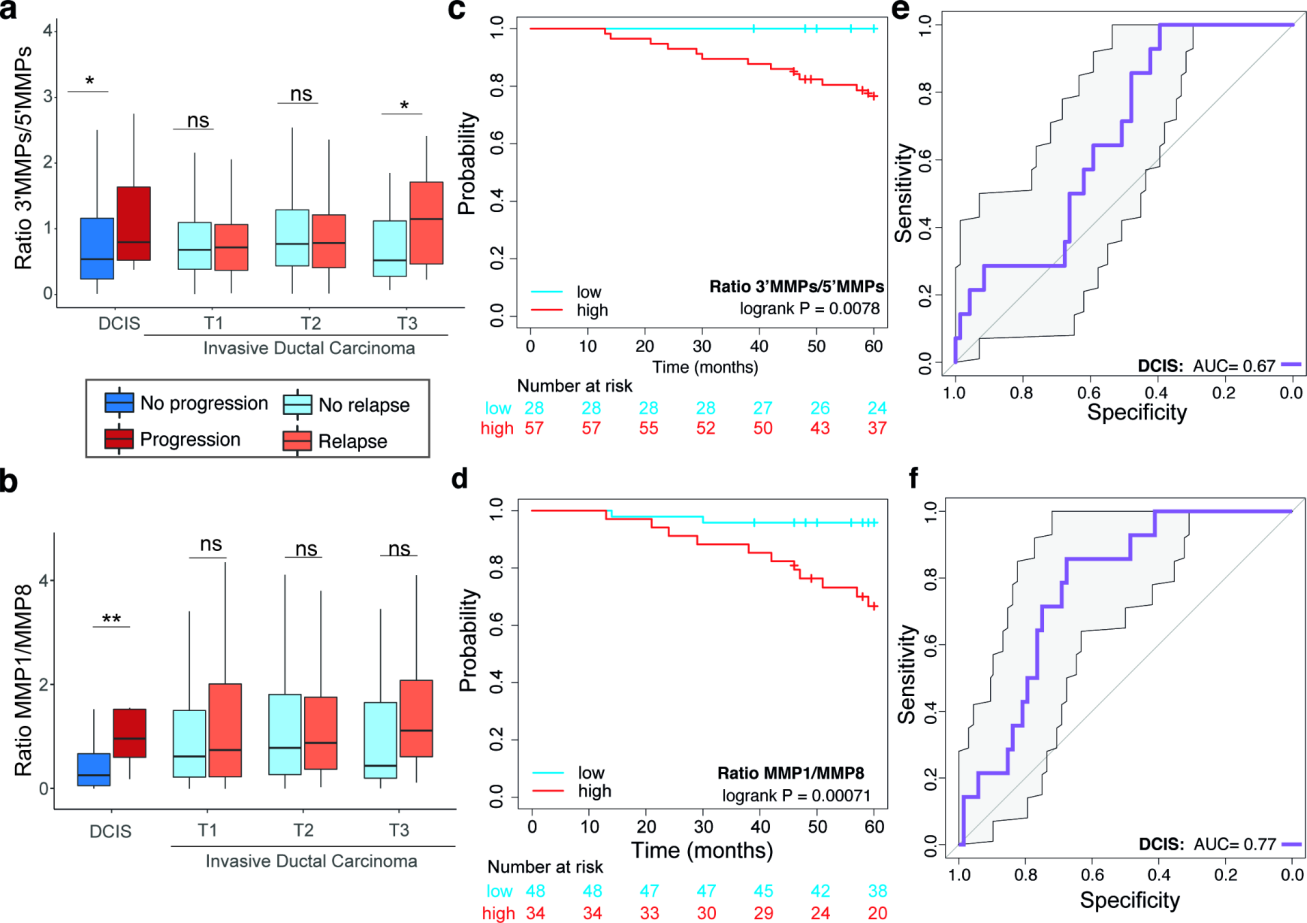
Implications of MMPs in DCIS progression. **(a)** 3’MMPs/5’MMPs and **(b)** MMP1/MMP8 ratios of standardized gene expression DCIS and invasive ductal carcinoma. Mann Whitney test. ns: no significant, **P < 0.01. Kaplan-Meier curves of relapse-free survival of DCIS using **(c)** 3’MMPs/5’MMPs and **(d)** MMP1/MMP8 ratios. ROC curves displaying the performance of **(e)** 3’MMPs/5’MMPs and **(f)** MMP1/MMP8 ratios in in situ ductal carcinoma

Finally, we explored whether the ratios between pro-invasive and antitumorigenic MMPs are linked to the progression of ductal carcinomas in situ (DCIS) to invasive ductal carcinoma (IDC) regardless of the breast cancer subtype using the transcriptomic and clinicopathological data from the SCAN-B cohort [31]. We compared clinical specimens of normal breast tissue, pure DCIS, and IDC from different TNM stages. The MMP (3’MMP/5’MMP) ratio was significantly higher in DCIS and IDC when compared to normal breast tissue (PDCIS < 0.001, PIDC < 0.001) (Figure S11a). Importantly, DCIS that eventually progressed to invasive disease displayed a significantly higher MMP ratio than those that did not progress (Fig. 6a). Remarkably when considering the ratio between the pro-invasive MMP1 and the antimetastatic MMP8, the differences in DCIS that progressed to invasive disease were even more pronounced (P = 0.002, Fig. 6b). There were no changes between IDC stratified by tumor size high MMP ratios were also associated with shorter relapse-free survival in patients with DCIS (p-value < 0.001) considering both 3’MMPs/5’MMPs and MMP1/MMP8 ratios (Fig. 6c and d). Importantly, both MMP ratios significantly predicted which DCIS patients will progress to invasive disease (AUC 3’MMPs/5’MMPs ratio = 0.67, AUC of MMP1/MMP8 = 0.77; Fig. 6e and f), but not, but not 3’MMPs and 5’MMPs signatures tested individually (Supplementary figures S11b). Moreover, we found that MMP1 is significantly upregulated in DCIS from patients who had ipsilateral breast events in an independent cohort of DCIS patients studied at the TBCRC 038 clinical trial [33] (Figure S11c).

## Discussion

Metastasis is an orchestrated process that starts with the escape of cancer cells from their primary niche and ends with the colonization of secondary sites. During the first stage, cancer cells degrade the basement membrane through the release and activation of MMP enzymes and undergo an EMT, establishing crosstalk with stromal cells [40]. In this study, we showed that the balanced expression profile of nine MMP genes located at chromosome 11 (Chr11q22.2) is influenced by an insulator element near the *MMP8* gene (IE8). CRISPR/Cas9-mediated disruption of IE8 triggered changes in chromatin accessibility and mRNA expression on the genomic region around IE8. Among these changes, we observed an upregulation of MMP8 and a downregulation of MMP1. This shift in mRNA expression was accompanied by differences in protein levels, activity, and most importantly, in the invasive properties of TNBC cells.

MMP1 upregulation displays a pivotal role in metastasis in several malignancies including TNBC [41]. MMP1 is overexpressed during lymph node metastasis in xenografted mice in a TNBC model and exosomes extracted from the serum of metastatic TNBC patients displayed higher levels of MMP1 than in patients with no metastatic disease [42]. In addition, RUNX2-mediated increment of MMP1 levels has been associated with chemoresistance [43]. Therefore, several publications support the role of MMP1 in aggressiveness in different malignancies. Specifically, on TNBC, Wang et al. observed a decrease in cell proliferation, migration, and colony formation after MMP1 knockdown through shRNAs in MDA-MB-231, one of the TNBC cell lines used in our study [12]. However, we did not observe these consequences after the IE8 disruption. Nevertheless, the ectopic expression of functional MMP1 triggered more pronounced differences, promoting enhanced clonogenic and migration abilities, when compared to IE8 disruption. This disparity could be explained by the fact that the decrease in MMP1 after IE8 disruption is not as pronounced as that seen with MMP1 mRNA-directed shRNAs. Beyond that, the cell line-dependent upregulation of MMP2 and MMP9 that we observe in our models may mitigate the impact of the reduction of MMP1 levels, an observation not reported by Wang et al. The effects on cell invasion after IE8 disruption detected in our study are consistent with the findings by Lim et al., who observed a decrease in invasiveness after the knockdown of the MMP1 upstream activating factor YBX1 [44].

The role of MMP8 in cancer is more controversial since its expression has been associated with both better and worse prognoses depending on the tissue of origin [15]. Most studies performed in breast cancer associate MMP8 with a protective role. MMP8-expressing cells are less invasive in vitro [45], systemic MMP8 expression decreases tumor size in mice [46] and MMP8 blood levels are associated with lower lymph node metastasis rates [14]. Various complementary studies delved into the underlying mechanisms that link MMP8 upregulation and the reported tumor-protective effects. MMP8 overexpression enhanced the cleavage of decorin, which diminished the transforming growth factor β (TGF-β) signaling. The decrease in this pathway promoted miR-21 downregulation and the subsequent induction of tumor suppressors such as programmed cell death 4 (PDCD4) [47]. MMP8 also alters the adhesive and proteolytic properties of the ECM, increasing cell-cell adhesion [48] and cleaving other MMPs, such as MMP3. We believe that the differential CTCF occupancy at IE8 may also contribute to the protective role that has been associated with *MMP8* upregulation since its activation – as well as the other 5’MMP genes – implies a compensatory decrease in *MMP1* and other 3’MMP genes. Thus, an integrative vision of MMPs encoded at the Chr11q22.2 rather than focusing on a single MMP may be more useful for determining the progression risk of breast cancer patients and evaluating potential therapeutic strategies.

The ratios of 3’MMPs/5’MMPs and MMP1/MMP8 were found to be higher in DCIS which eventually progressed to invasive disease (Fig. 6). In fact, MMP1 has been previously associated with DCIS with micro-invasive foci [49], whereas MMP8 loss has been linked to DCIS progression [48]. Interestingly, the MMP1/MMP8 ratio alone exhibited similar performance to the HTAN DCIS classifier generated by Strand et al. [33] which was trained in the TBCRC 038 cohort (AUC = 0.72 in the RAHBT validation cohort). Therefore, this alteration appears to be relevant during the invasive transition of this disease. While further studies are needed to characterize the role of IE8 activation in breast cancer invasion, these results point towards a potential dynamic regulation of the gene expression program at Chr11q22.2.

Genome-wide analyses have revealed a strong overlap between chromatin loops and CTCF binding sites [50]. Furthermore, different studies have proven that the alteration of the CTCF binding site – either through its disruption or its inversion – has an impact on chromatin architecture, which disturbs promoter-enhancer interactions [51, 52]. Moreover, the gain or loss of cancer-specific CTCF binding events contributes to oncogenic transcriptional programs [53]. CTCF binding ability can be impaired through somatic mutations [21], but also DNA methylation [54]. We characterized that CTCF is effectively bound to the CTCF binding site located at IE8 in our TNBC models. After IE8-disruption and consequent CTCF decoupling, gene regulatory elements on both 5’ and 3’MMP regions can physically interact again. Consequently, IE8-disruption is followed by an increase in chromatin accessibility on promoter regions of the 5’MMP region as well as higher exposure of enhancer elements of both the 5’ and 3’MMP regions. The interplay between IE8 and the expression of local MMPs is not restricted to our cell models. We observed a strong positive correlation between chromatin accessibility at IE8 and the expression ratio between 3’MMPs (MMP1, MMP3, MMP10, MMP12, MMP13) and 5’MMPs (MMP7, MMP8, MMP20, MMP27) in TNBC patients, supporting the idea that IE8 effectively contributes to the modulation of gene expression at the Chr11q22.2 in this malignant neoplasm (Figures S8e and S8f).

In summary, we combined multi-omics profiling with functional experiments to characterize the regulation of MMPs encoded at Chr11q22.2. This study provides evidence that a single chromatin insulator located between MMP8 and MMP10 orchestrates the expression of two clusters of MMP genes in TNBC, which are associated with invasiveness and whose expression profile appears to impact DCIS progression and survival outcomes in patients with breast cancer.

### Electronic supplementary material

Below is the link to the electronic supplementary material.

Supplementary Material 1. It includes supplementary figures, table S1, and extended methods.

## Data Availability

All raw and processed data are freely available from the ENA repository and have been deposited under the following accession codes: E-MTAB-12,825 (Hi-C), E-MTAB-12,821(ATAC-seq), E-MTAB-12,823 (RNA-seq). A detailed explanation of the generation and usage guidelines for these datasets is available in the related data descriptor manuscript [55].

## Code Availability

All analyses were performed using open-source R and Python packages. ATAC-seq read processing was performed using the following software versions: nf-core/atacseq (v1.2.1), Nextflow (v21.04.3), FastQC (v0.11.9), Trim Galore!(v0.6.4_dev), BWA (v0.7.17-r1188), Samtools (v1.10), BEDTools (v2.29.2), BamTools (v2.5.1), deepTools (v3.4.3), Picard (v2.23.1), R (v3.6.2), Pysam (v0.15.3), MACS2 (v2.2.7.1), ataqv (v1.1.1), featureCounts (v2.0.1), Preseq (v2.0.3), MultiQC (v1.9). HiC read processing was performed using the following software versions: nf-core/hic (v1.3.0), Nextflow (v21.10.6), Bowtie2 (v2.3.5.1), Python (v3.7.6), Samtools (v1.9), MultiQC (v1.8). RNA-seq reads processing was performed using the following software versions: bcl2fastq (v2.20.0.422), FastQC (v0.11.9), MultiQC (v1.13.dev0), Fastp (v0.21.0), HISAT2 (v2.2.0), Samtools (v1.10), StringTie (v2.1.4). DESeq2 (1.3.1) was used to calculate differentially expressed genes from RNA-seq and perform PCA analysis. Further data processing was performed using R packages from tydiverse, such as dplyr, tidyr, and ggplot2; pheatmap, rstatix, and viridis. Galaxy servers were also used for ATAC-seq analysis.

## List of abbreviations

ATAC-seq: Assay for Transposase-Accessible Chromatin using sequencing
ATCC: American Type Culture Collection
AUC: Area under the curve
CCLE: Cancer Cell Line Encyclopedia
ChiA-PET: Chromatin Interaction Analysis with Paired-End Tag
CTCF: CCCTC-Binding Factor
CUT&RUN: Cleavage Under Targets and Release Using Nuclease
DCIS: Ductal carcinoma In situ
DEGs: Differentially Expressed Genes
ECM: Extracellular Matrix
EE: Enhancer element
EMT: Epithelial-to-Mesenchymal Transition
eQTLs: Expression of quantitative trait loci
EV: Empty vector
FBS: Fetal bovine serum
GO: Gene ontology
GTEX: Genotype-Tissue Expression
Hi-C: High throughput chromosome conformation capture
HR: Hazard ratio
IDC: Invasive Ductal Carcinoma
IE: Insulator element
IE8: Insulator element close to MMP8
Indel: Insertion or deletion
MMP: Matrix metalloproteinase
OS: Overall survival
PCAWAG: Pan-Cancer Analysis of Whole Genomes
qPCR: Quantitative PCR
RFS: Relapse-Free survival
RIN: RNA Integrity Number
RNA-seq: RNA sequencing
sgRNA: Single guide RNA
SNP: Single Nucleotide Polymorphism
SNV: Single Nucleotide Variant
T: Tumor size
TAD: Topologically Associating Domain
TCGA: The Cancer Genome Atlas
TIMP: Tissue Inhibitor of Metalloproteinases
TNBC: Triple-negative breast cancer
TSS: Transcription start site
WT: Wildtype
