# Supplementary material for: Chromatin insulation orchestrates matrix metalloproteinase gene cluster expression reprogramming in aggressive breast cancer tumors
Source: Mol Cancer. 2023 Nov 28;22:190. doi: 10.1186/s12943-023-01906-8 (PMC10683115; doi:10.1186/s12943-023-01906-8)
Supplement: Supplementary file 1 — Supplementary Material 1. It includes supplementary figures, table S1, and extended methods. [file 12943_2023_1906_MOESM1_ESM.docx]

**Chromatin insulation orchestrates matrix metalloproteinase gene cluster expression reprogramming in aggressive breast cancer tumors**

Pere Llinàs-Arias^1^, Miquel Ensenyat-Mendez^1^, Sandra Íñiguez-Muñoz^1^, Javier I. J. Orozco^2^, Betsy Valdez^2^, Matthew P. Salomon^3^, Chikako Matsuba^3^, Maria Solivellas-Pieras^1^, Andrés F. Bedoya-López^1^, Borja Sesé^1^, Anja Mezger^4^, Mattias Ormestad^4^, Fernando Unzueta^5^, Siri H. Strand^6^, Alexander D. Boiko^7^, E Shelley Hwang^8^, Javier Cortés^9,10,11^, Maggie L. DiNome^8^, Manel Esteller^12,13,14,15^, Mathieu Lupien^16,17,18^ and Diego M. Marzese^1,8*^

**Affiliation**

1. Cancer Epigenetics Laboratory, Health Research Institute of the Balearic Islands (IdISBa), 07120 Palma, Spain.

2. Saint John's Cancer Institute, Providence Saint John's Health Center, Santa Monica, CA, USA.

3. USC Research Center for Liver Diseases, Keck School of Medicine, University of Southern California, Los Angeles, CA, USA

4. Science for Life Laboratory, 17665 Solna, Sweden.

5. Advanced Optical Microscopy Facility Scientific and Technological Centres of University of Barcelona, Barcelona, Spain.

6. Department of Pathology, Stanford University School of Medicine, Stanford, CA 94305, USA.

7. Cedars-Sinai Medical Center, Samuel Oschin Comprehensive Cancer Institute, Department of Medicine, Los Angeles, CA 90048, USA

8. Department of Surgery, Duke University School of Medicine, Durham, NC, USA.

9. International Breast Cancer Center (IBCC), Pangaea Oncology, Quiron Group, 08017 Barcelona, Spain.

10. Medica Scientia Innovation Research SL (MEDSIR), 08018 Barcelona, Spain.

11. Department of Medicine, Faculty of Biomedical and Health Sciences, Universidad Europea de Madrid, 28670 Madrid, Spain.

12. Josep Carreras Leukaemia Research Institute, Badalona, Barcelona, Catalonia, Spain.

13. Centro de Investigación Biomédica en Red Cancer (CIBERONC), 28029, Madrid, Spain.

14. Institució Catalana de Recerca i Estudis Avançats (ICREA), Barcelona, Catalonia, Spain.

15. Physiological Sciences Department, School of Medicine and Health Sciences, University of Barcelona (UB), Barcelona, Catalonia, Spain.

16. Princess Margaret Cancer Centre, Toronto, Ontario M5G 1L7, Canada.

17. Department of Medical Biophysics, University of Toronto, Toronto, Ontario M5G 1L7, Canada.

18. Ontario Institute for Cancer Research, Toronto, Ontario M5G 0A3, Canada.

***Corresponding Authors Information:**

Diego M. Marzese, PhD

Cancer Epigenetics Laboratory

Institut d'Investigació Sanitària Illes Balears

Palma, Balearic Islands, 07120 Spain.

+34 (871) 205-234

Email: [diego.marzese@ssib.es](mailto:diego.marzese@ssib.es)

**Keywords:** MMP1, MMP8, CTCF, insulator, chromatin, gene regulatory element, cis-regulatory element, breast cancer, invasion, ATAC-seq, RNA-seq, Hi-C

### **SUPPLEMENTARY FIGURES**


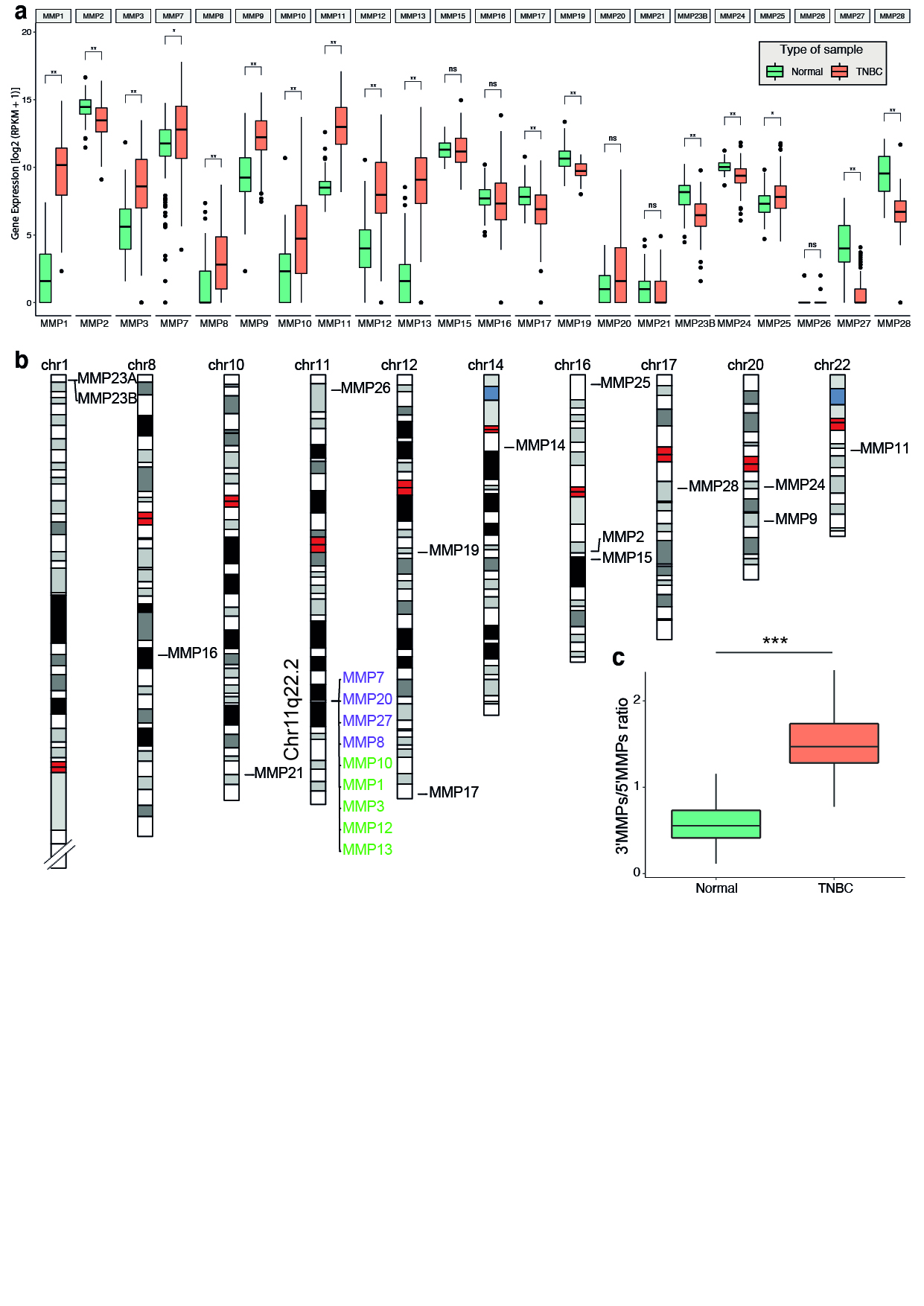


**Supplementary Figure 1. Related to Figure 1. a.** Gene expression of all human MMP genes in normal and TNBC samples. **b.** MMP gene distribution across chromosomes. **c.** The ratio of the 3’MMPs/5’MMPs between normal and TNBC samples. Mann-Whitney test. ns: no significant, *P<0.05, **P<0.01, ***P<0.001.


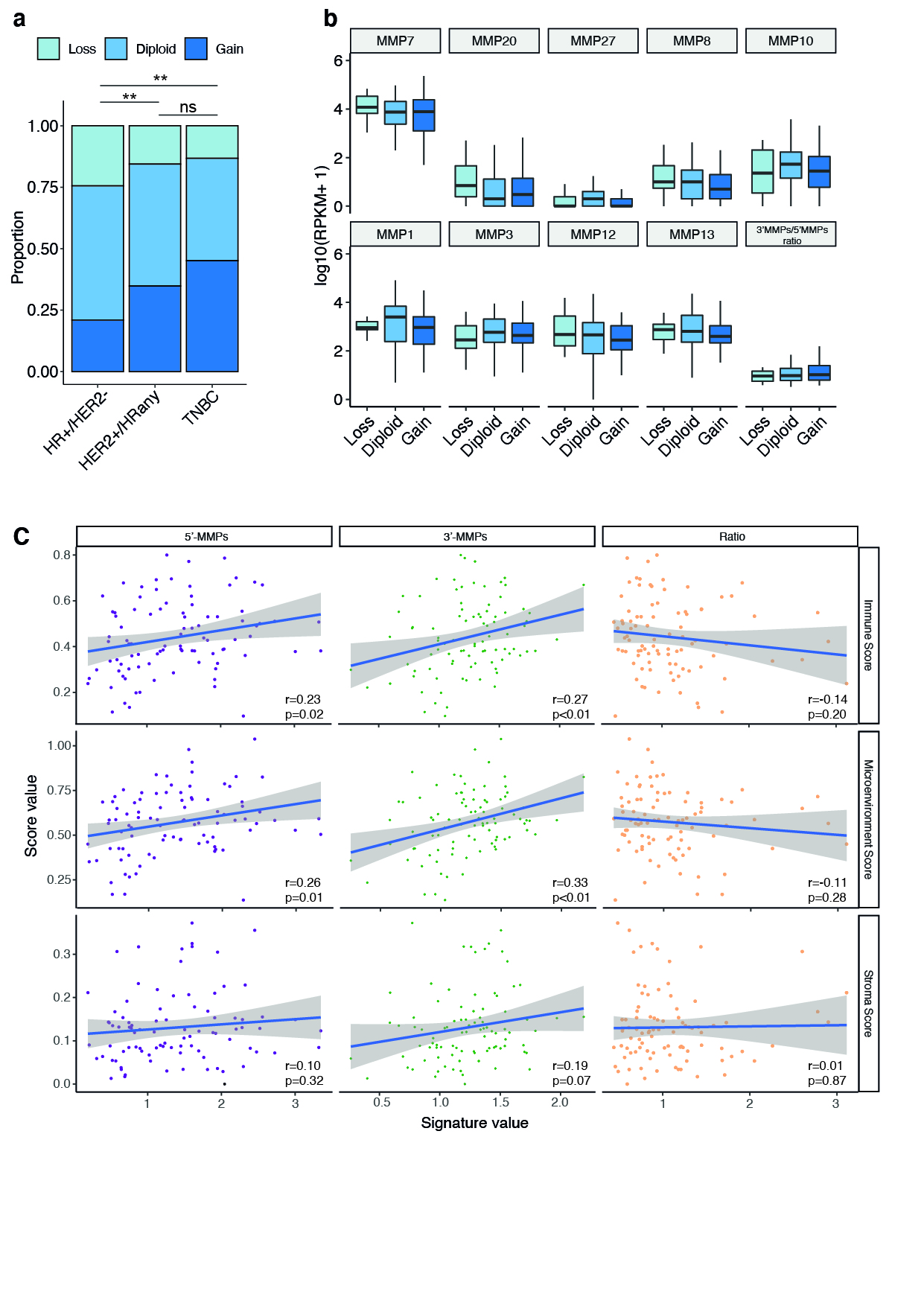


**Supplementary Figure 2. Related to Figure 1.** a. Copy number analysis for the MMP locus (1 Mb) on different breast cancer subtypes. Loss: < 2 copies, diploid: 2 copies, and gain: > 2 copies. **b.** Expression levels of MMP genes encoded at Chr11q22.2 in TNBC samples according to the copy number of the Chr11q22.2 region. **c.** Pearson’s correlation analysis between MMP signatures (5’MMPs, 3’MMPs, and 3’MMPs/5’MMPs ratio) and cell heterogeneity scores (Inmuno Score, Microenvironment Score, and Stroma Score) calculated through transcriptomic deconvolution.

**
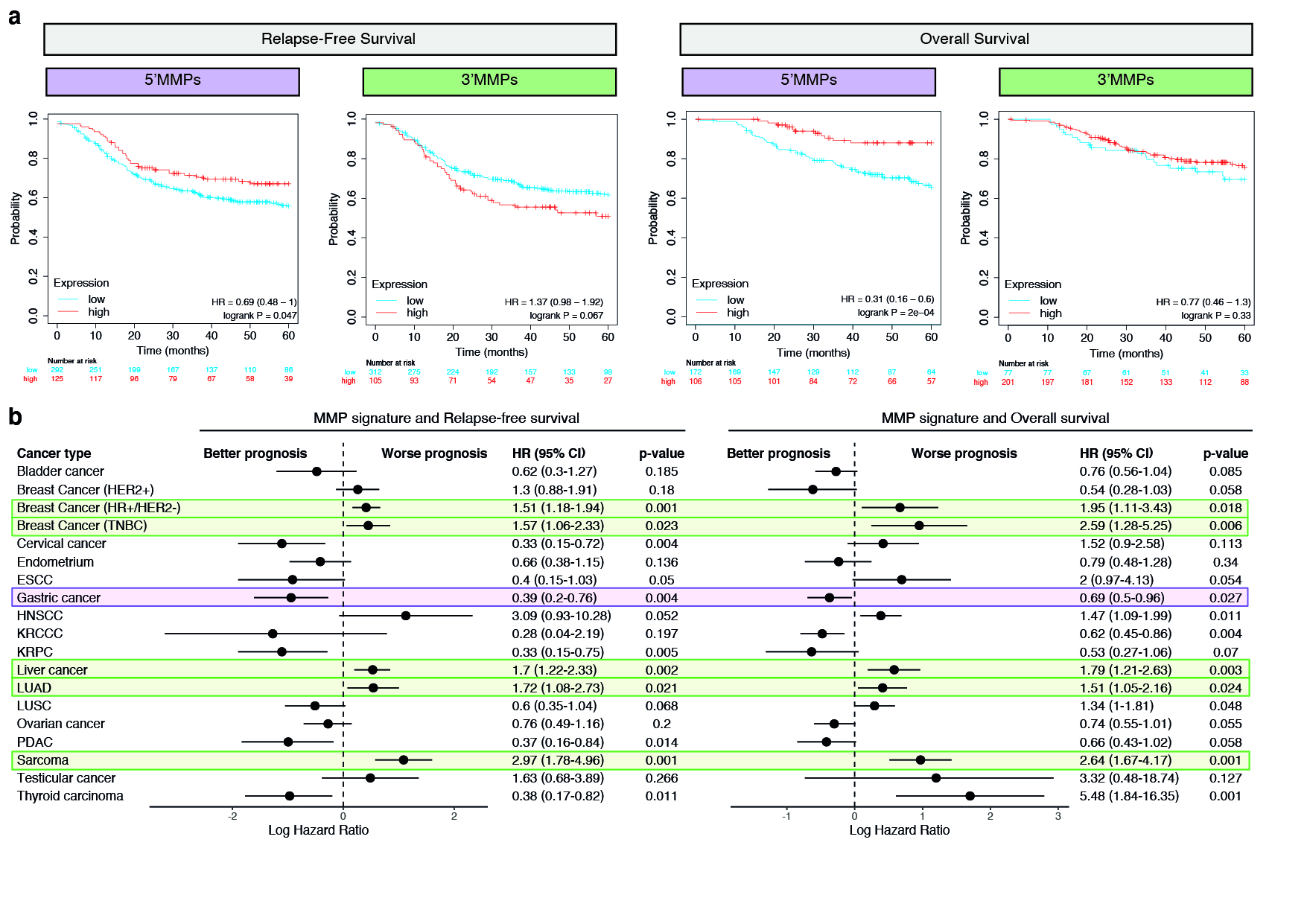
**

**Supplementary Figure 3. Related to Figure 1.** **a.** Kaplan-Meier curves depicting relapse-free survival and overall survival according to the expression of 5’MMP genes alone (purple) or 3’MMP genes alone (green). **b.** Forest plots displaying the Log2 Hazard ratio for RFS (left) and OS (right) of high 3’MMP/5’MMP ratio signature in different cancer types. Tumor subtypes and cancer types where the 3’MMP/5’MMP ratio is associated with a worse prognosis are highlighted in green, whereas Gastric cancer, where the 3’MMP/5’MMP ratio is associated with a better prognosis is highlighted in purple. HR+: hormone receptor-positive; ESCC: esophageal squamous cell carcinoma; HNSCC: head and neck squamous cell carcinoma; KRCCC: kidney renal clear cell carcinoma; KRPC: kidney renal papillary cell carcinoma; LUAD: Lung adenocarcinoma; LUSC: Lung squamous cell carcinoma; PDAC: Pancreatic ductal adenocarcinoma.

**
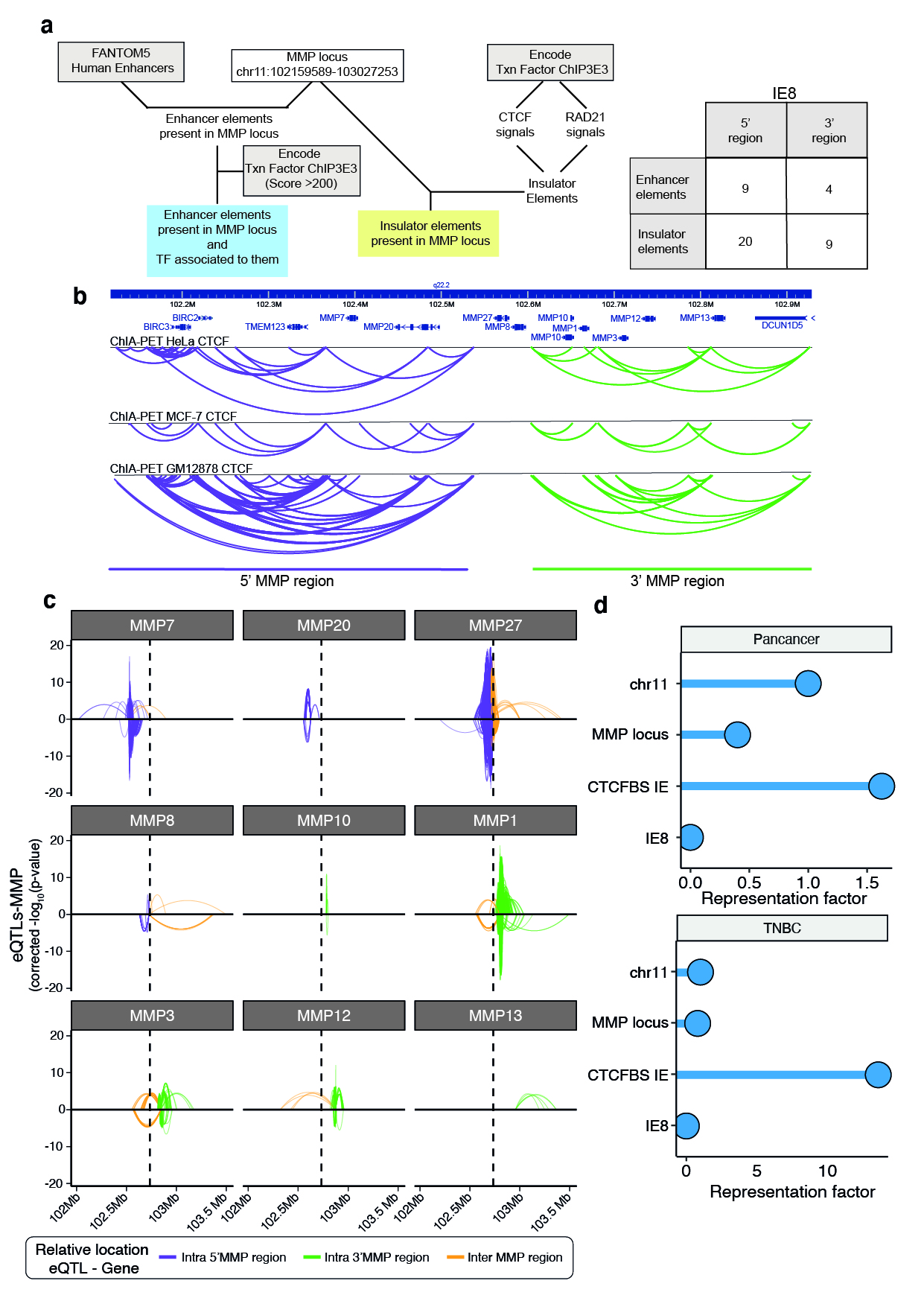
**

**Supplementary Figure 4. Related to Figure 2. a.** Workflow describing the gene regulatory elements identification (**left**) and summary of characterized gene regulatory elements by spatial location (**right**). **b.** ChIA-PET against CTCF at Chr11q22.2 in different cell lines. **c.** Representation of QTLs influencing the expression (eQTIL) of MMP genes at Chr11q22.2. Arc colors summarize the interactions according to their relative location. Purple arcs link SNPs and TSS located at the 5’MMP locus, green arcs connect both SNPs and TSS found at the 3’MMP locus and orange arcs correspond to interactions that cross IE8, which is indicated by a dashed vertical line. Arc height is represented by -log_10_(p-value) for eQTL that are associated with an increase in expression and log_10_(p-value) for eQTL is linked to a decrease in expression. **d.** Representation factor of SNPs located at MMP locus, CTCF binding sites of Insulator Elements, and IE8. All data are normalized to chr11 mutation considering the length (bp) of segments. Lollipops also include the raw number of mutations.


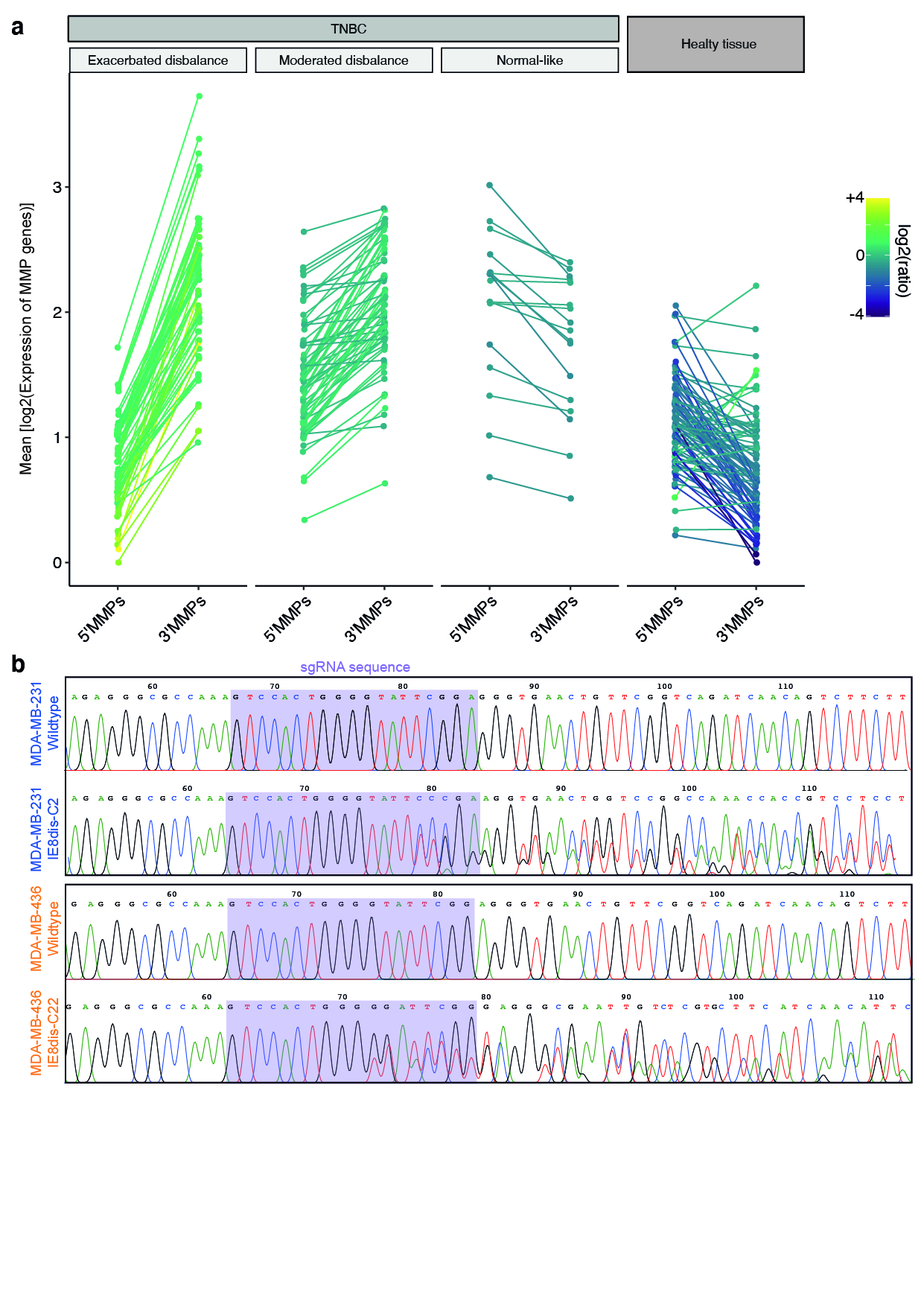


**Supplementary Figure 5. Related to Figure 2. a.** Expression profile of 5’ and 3‘MMP genes in Normal breast tissues and TNBC tissues from TCGA. **b.** Electropherograms of the sgRNA surrounding sequence (**highlighted in purple**) in MDA-MB-231 and MDA-MB-436 wildtype and the selected IE8-disrupted clones.


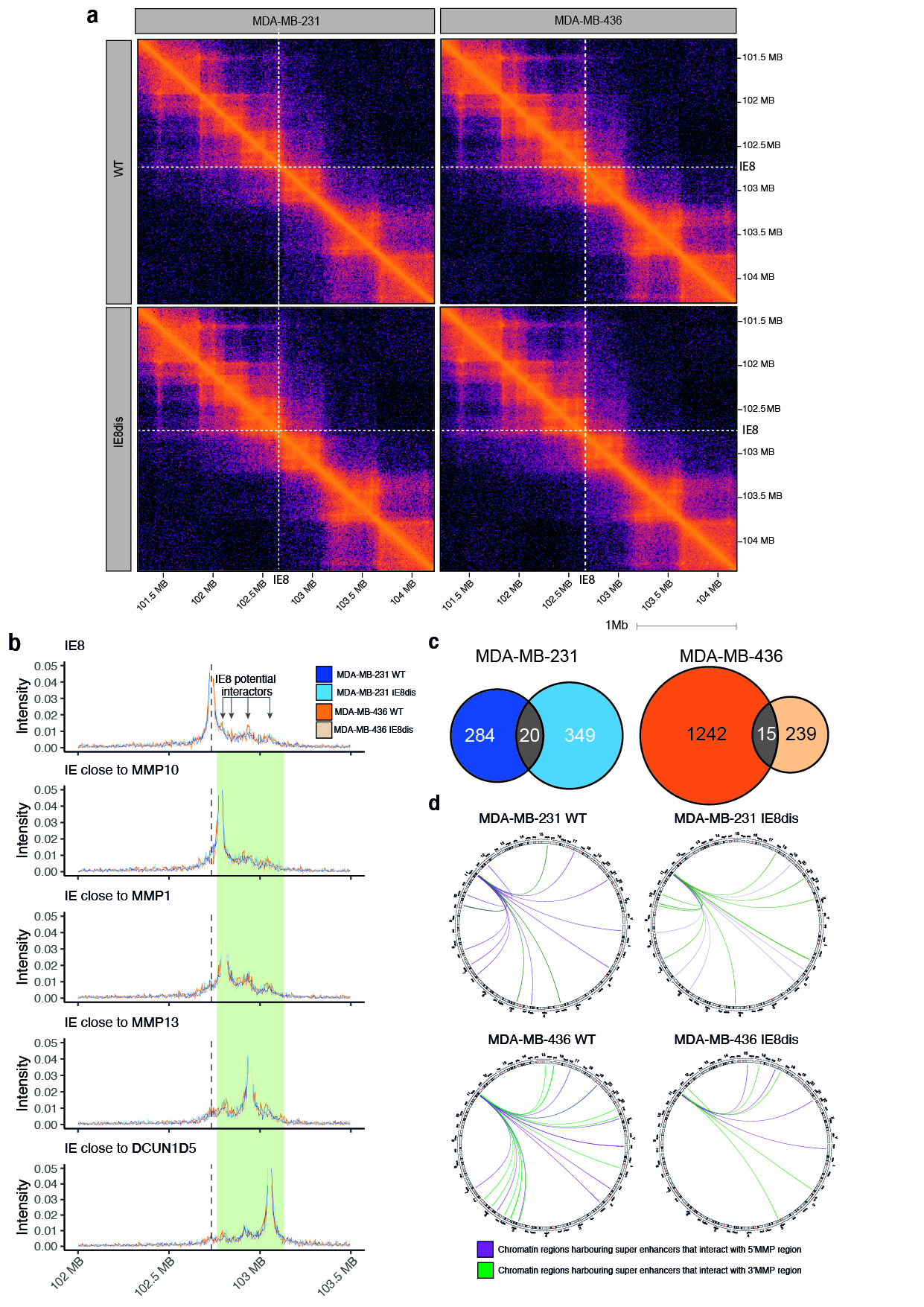


**Supplementary Figure 6. Related to Figure 2. a.** Hi-C contact frequency matrix for the 3Mb genomic region surrounding the IE8 binned at 10-kb resolution in MDA-MB-231 and MDA-MB-436 before and after IE8 disruption. **b.** The intensity of contact interactions of IE8 and its potential IE partners. **c.** Characterization of high-confidence interchromosomal interactions in MDA-MB-231 and MDA-MB-436. **d.** Interchromosomal interactions between the 5’-end (purple ribbons) and 3’-end (green ribbons) of Ch11q22.2 and super-enhancer elements.


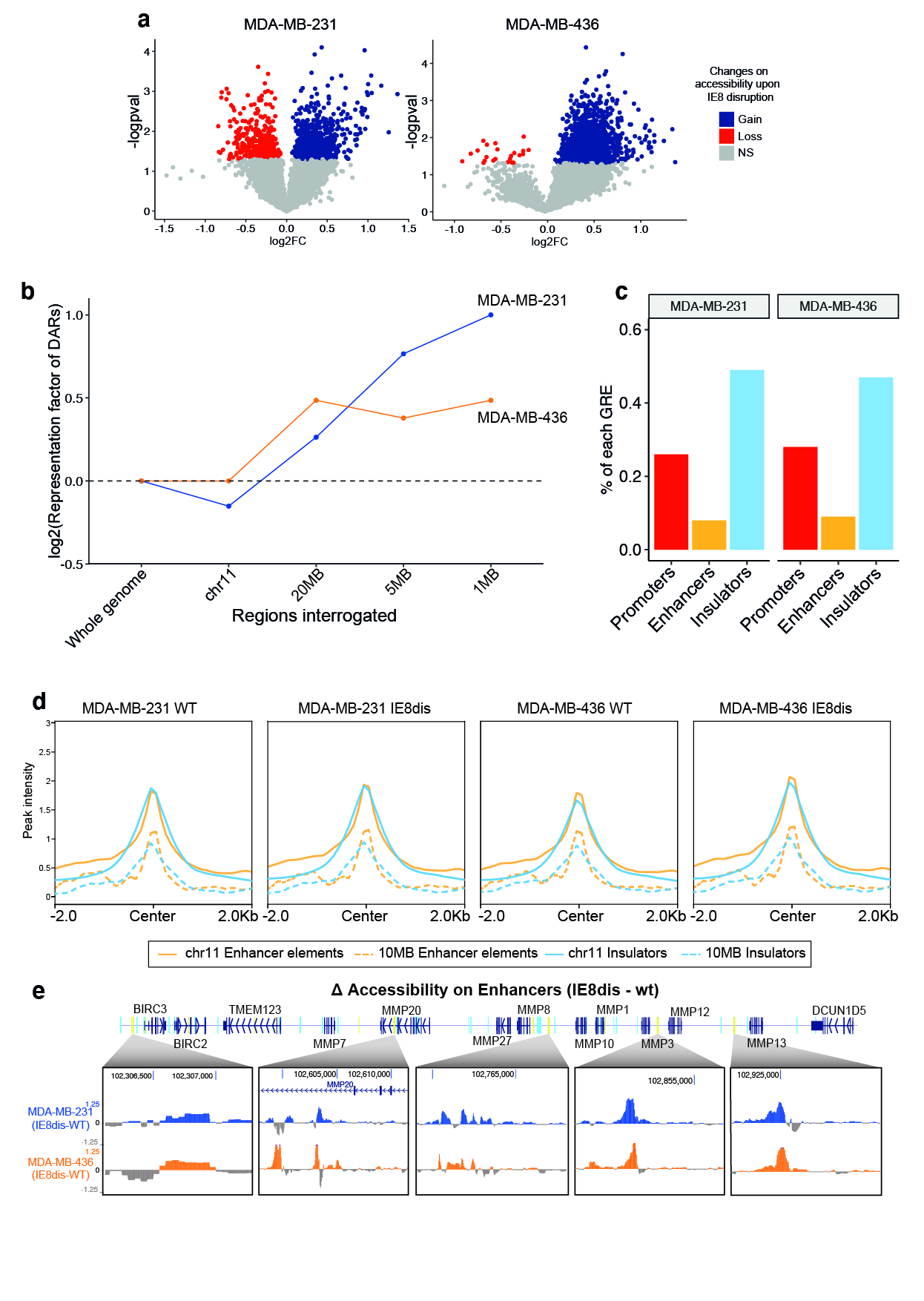


**Supplementary Figure 7. Related to Figure 3. a.** Volcano plots of overlapping peaks between WT and IE8dis conditions for MDA-MB-231 and MDA-MB-436 cell models. **b.** Fold change of the representation factor of differentially accessible regions (DARs) detected on each cell line model. **c.** Gene regulatory element assignment of differential peaks. **d.** Peak intensity stacked of enhancer elements and insulators located on Chr11 and 10Mb around the IE8. **e.** Illustrative examples of the variation in chromatin accessibility at enhancer elements located at the MMP locus after IE8 disruption in MDA-MB-231 and MDA-MB-436.


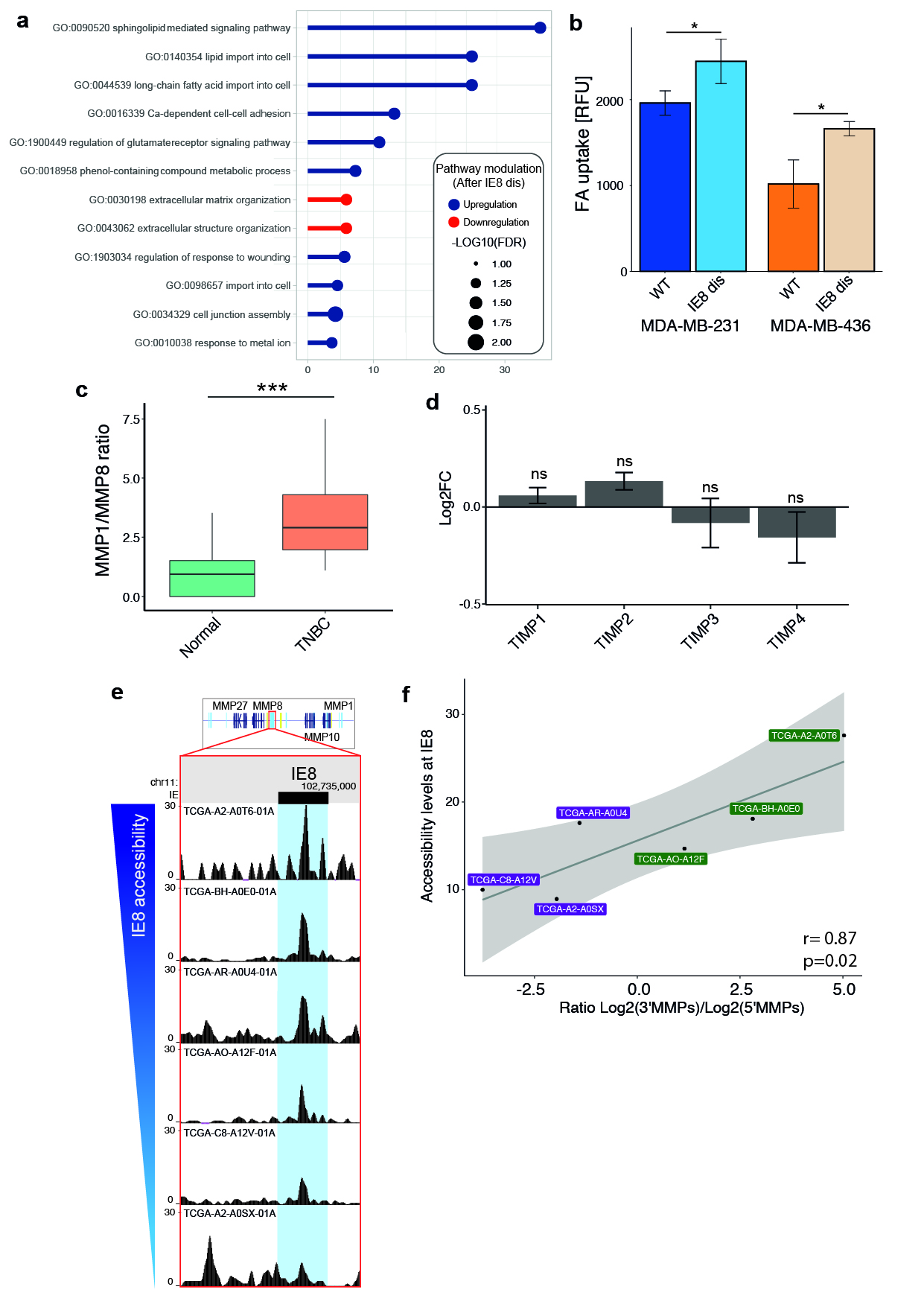


**Supplementary Figure 8. Related to Figure 4. a.** Gene Ontology results for the most enriched biological processes according to the differentially expressed genes upon IE8 disruption. **b.** Bar plot describing fatty acid uptake assessed 15 min after incubation of MDA-MB-231 and MDA-MB-436 before and after IE8 disruption with fatty acid uptake reagent. **c.** The ratio between MMP1/MMP8 comparing normal and TNBC samples. Mann-Whitney test. ***P<0.001. **d.** Differential expression of TIMP1, 2, 3, and 4 genes before and after IE8 disruption using the RNA-seq from the MDA-MB-231 model. ns: non-significant. **e.** Chromatin accessibility was determined as peak intensity on TCGA TNBC samples **f.** Pearson’s correlation between IE8 accessibility and the ratio between the log2 expression of 3’MMPs and 5’MMPs.


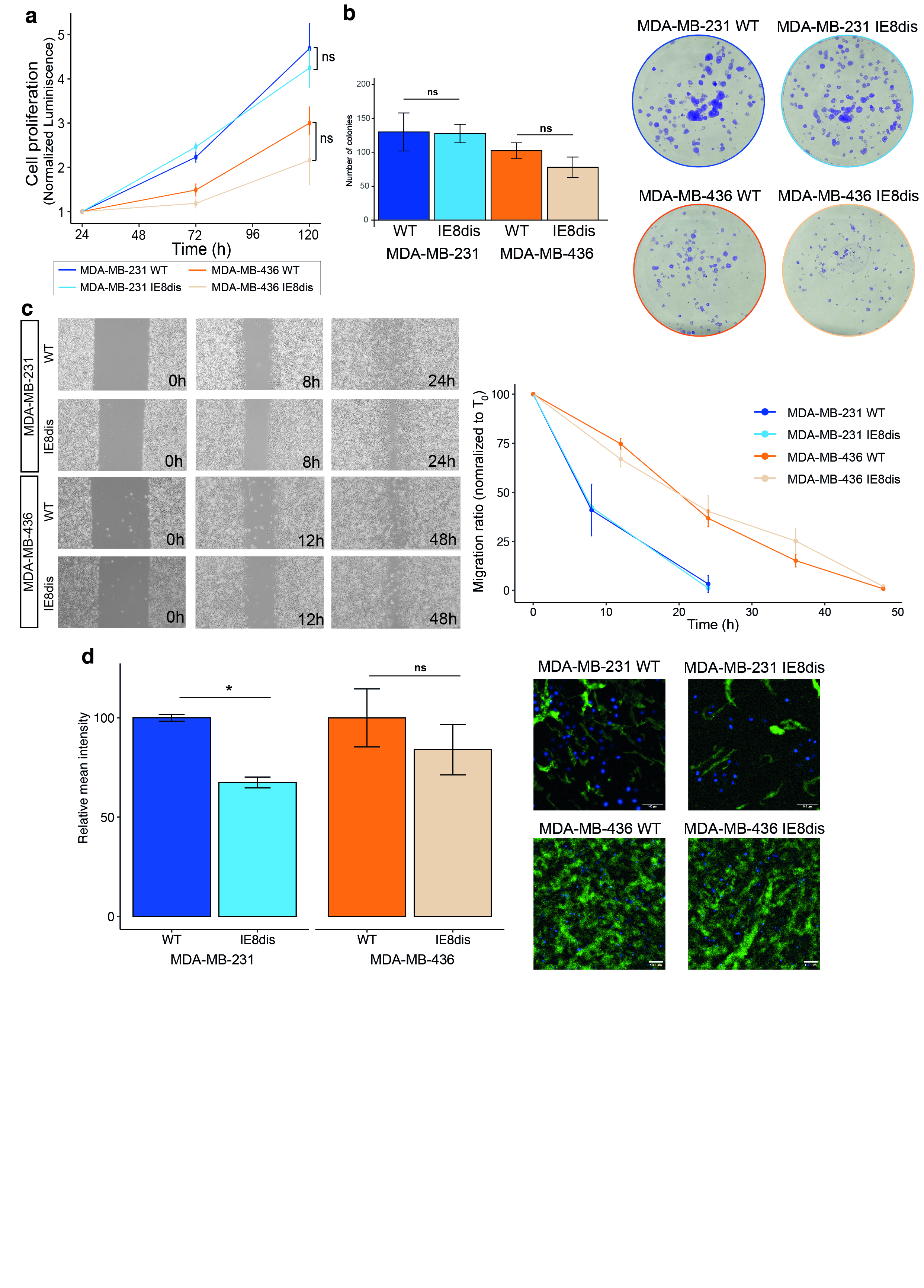


**Supplementary Figure 9. Related to Figure 5. a.** Time-course of cell proliferation based on ATP levels detected by luminescence using CellTiter Glo before and after IE8 disruption in MDA-MB-231 and MDA-MB-436 cells. **b.** Number of colonies (**left**) and illustrative examples generated from 200 cells from MDA-MB-231 and MDA-MB-431 before and after IE8 disruption (**right**). **c.** Migration ability was assessed by wound-healing assay before and after the IE8 disruption on MDA-MB-231 and MDA-MB-431. (**left**) Illustrative images of wound-healing migration assay. (**right**) Wound healing quantification. **d.** (**left**) quantification of the collagen type-I fiber degradation ability before and after IE8 disruption using DQcollagen (**right**) illustrative images of fluorescence signal released after collagen type-I fiber degradation.


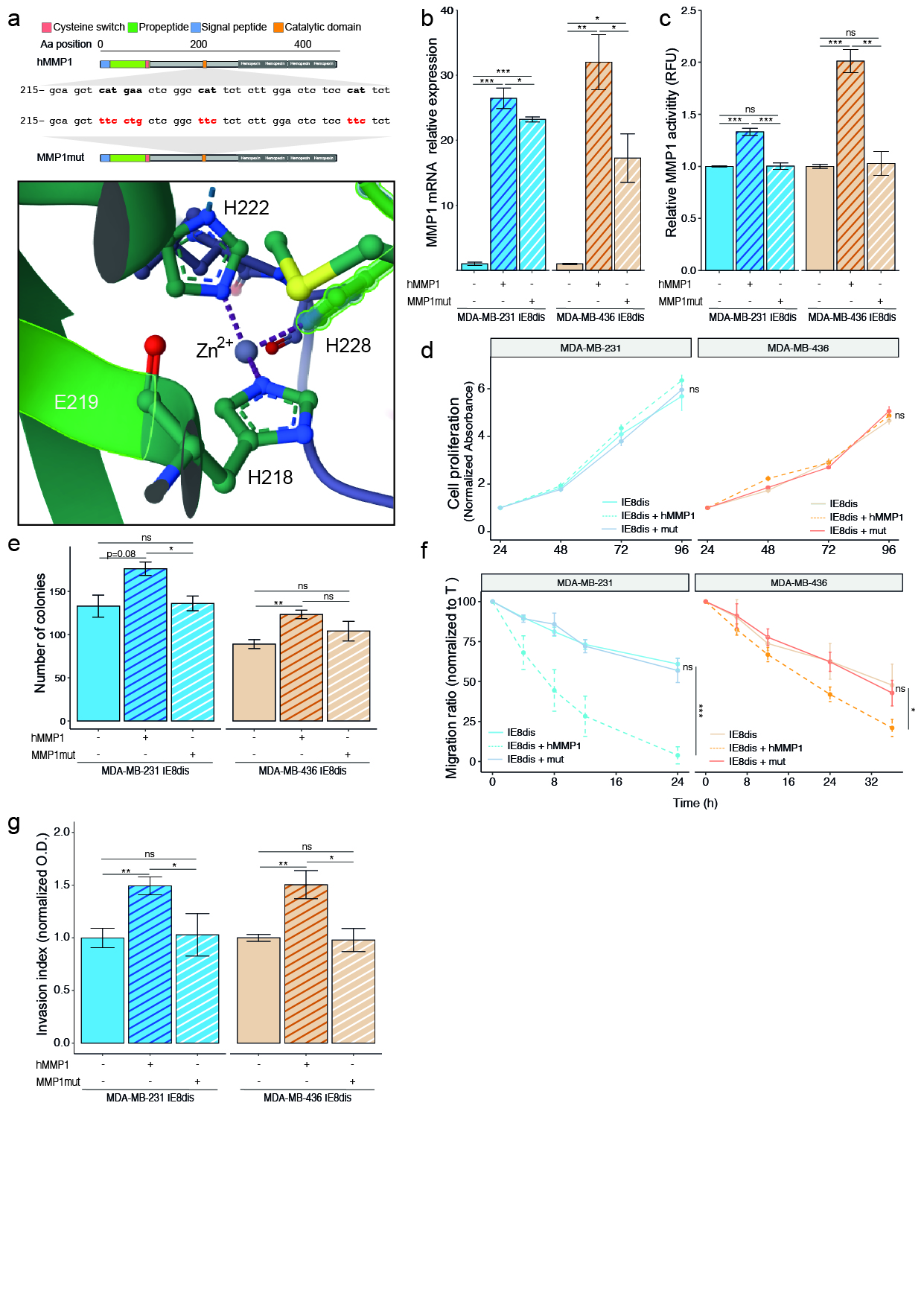


**Supplementary Figure 10. Related to Figure 5. a. (top)** Modifications on the gene sequence encoding the MMP1 catalytic domain to generate a catalytic defective variant (MMP1mut). (**bottom**) Amino acids that were replaced from the wild-type version of MMP1 were highlighted in the 3D MMP1 predicted structure. **b.** MMP1 mRNA expression levels and **c.** MMP1 activity in MDA-MB-231 and MDA-MB-436 IE8 disrupted after hMMP1 or MMP1mut ectopic expression. **d.** Time-course of cell proliferation in MDA-MB-231 and MDA-MB-436 with disruption of the IE8 with the ectopic expression of hMMP1 or MMP1mut. **e.** The number of colonies generated from 200 cells in both cell lines comparing the hMMP1 or MMP1mut ectopic expression on IE8 disrupted cells. **f.** Wound healing migration ratio on MDA-MB-231 and MDA-MB-431. **g.** Colorimetric quantification of cell invasion MDA-MB-231 and MDA-MB-436 cell invasion. Student’s T-test. ns: no significant, *P<0.05, **P<0.01, ***P<0.001. O.D. Optical density.


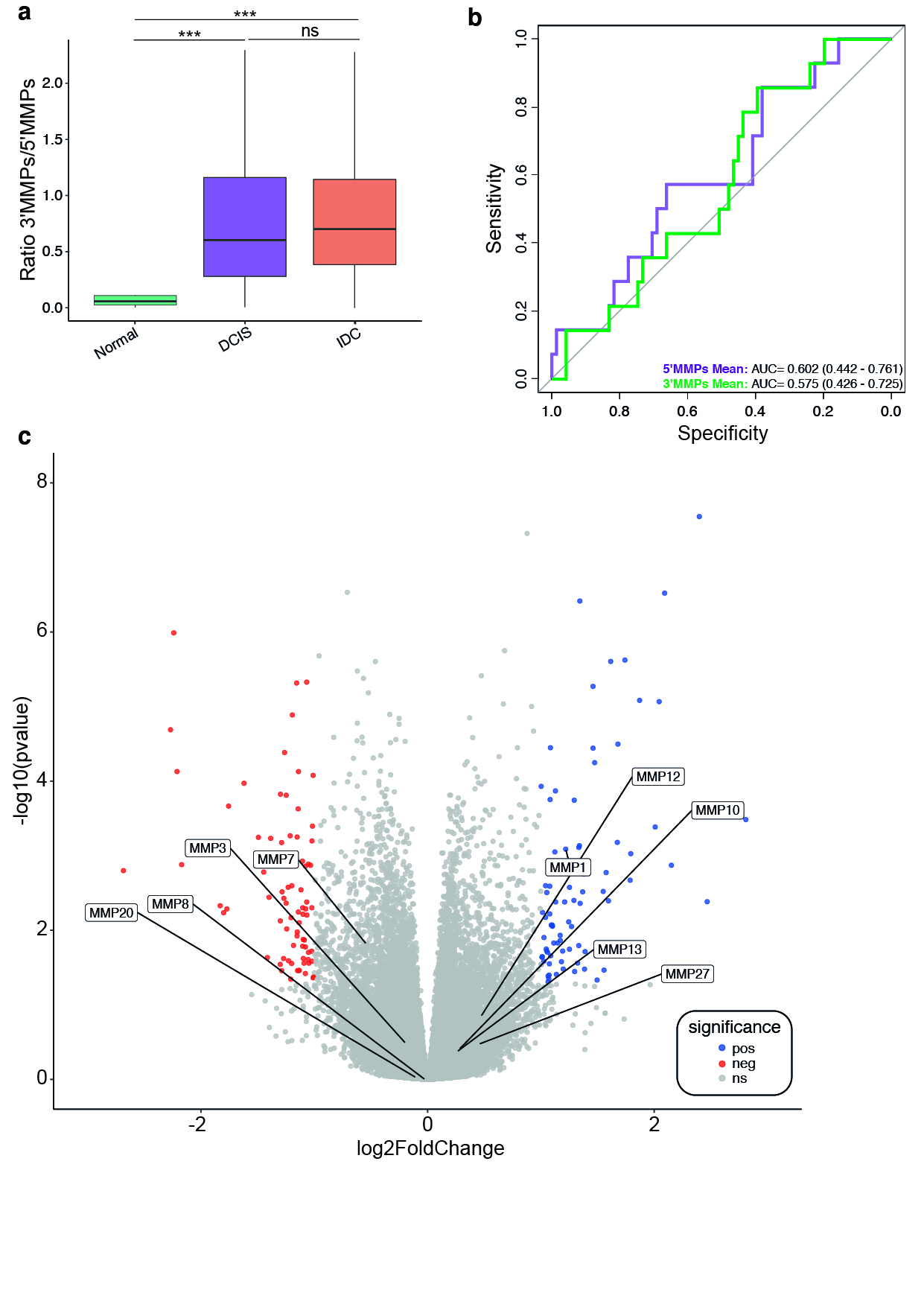


**Supplementary Figure 11. Related to Figure 6. a.** 3’MMPs/5’MMPs ratio comparing normal breast samples with ductal carcinoma in situ (DCIS) and invasive ductal carcinoma (IDC). **b.** ROC curves displaying the performance to predict progression of 5’ MMPs and 3’MMPs signatures tested individually **c.** Volcano plot showing differentially expressed genes between DCIS with and without an ipsilateral breast event (DCIS or invasive breast cancer; restricted to 5 years of follow-up) in the TBCRC 038 cohort. MMPs encoded in Chr11q22.2 appear highlighted.

**Supplementary tables**

**Table S1.** List of primers used in sgRNA design, amplicon generation, amplicon sequencing, qPCR from enriched chromatin, and qPCR from RNA-derived cDNA.

| Technique | Name | Sequence |
| --- | --- | --- |
| sgRNA cloning | sgRNA_Fd | CACCGTCCACTGGGGTATTCGGA |
|  | sgRNA_Rv | AAACTCCGAATACCCCAGTGGAC |
| Amplicon containing sgRNA for sequencing verification | Amplicon_IE8_Fd | CCCTACTTCAGTCCCCTCT |
|  | Amplicon_IE8_Rv | TTCAAAACCTGGGCTCATCC |
| Sanger sequencing | Amplicon_IE8_seq | TTCAATGAAGTCACGGATGG |
| CUT&RUN | IE8_r2_Fd | TGCTAGTGGTCATCATGCCC |
|  | IE8_r2_Rv | AGTTCACCCTCCGAATACCC |
| qPCR | qGAPDH_Fd | TGCACCACCAACTGCTTAGC |
|  | qGAPDH_Rv | GGCATGGACTGTGGTCATGAG |
|  | qMMP1_Fd | CCCACAAACCCCAAAAG |
|  | qMMP1_Rv | GATTTGTGCGCATGTAGAATCT |
|  | qMMP8_Fd | AGTTTTCCAGCAAGAACATT |
|  | qMMP8_Rv | CAGTTAAGCCATTTATTGCC |
|  | qMMP2_Fd | GATAACCTGGATGCCGTCGT |
|  | qMMP2_Rv | CGAAGGCAGTGGAGAGGAAG |
|  | qMMP9_Fd | ATGCCATTCACGTCGTCCTT |
|  | qMMP9_Rv | TGACAGCGACAAGAAGTGGG |

**Extended methods**

**Generation of stable models.** Single guide RNA (sgRNA) against the CTCF binding site located on the 3’-side of the MMP8 gene (chr11:102,730,781-102,736,005) was designed using the CHOPCHOP web tool (https://chopchop.cbu.uib.no/) [1]. sgRNA was assembled and cloned into a PsPCas9pSpCas9(BB)-2A-GFP (PX458) as described by Ran, Fa et al [2]. PX458 was a gift from Feng Zhang lab (Addgene # 48138). TNBC cell lines were transiently transfected with either the sgRNA-containing plasmid or the empty vector (EV) using lipofectamine 3000 (Thermofisher), following the manufacturer’s instructions. Two days after transfection, the cells were sorted by FACS, and the EV-transfected GFP-positive cells were collected and maintained as the wild-type population. On the other hand, GFP-positive cells transfected with sgRNA-containing plasmid were serially diluted in 96-well plates to isolate single clones. The disruption of the sgRNA target sequence was analyzed by Sanger sequencing (Macrogen, Madrid). Briefly, clones were grown, and DNA was purified using the Quick-DNA Microprep Kit (Zymo research). DNA containing the sgRNA target sequence was amplified by PCR, purified using NucleoSpin Gel and PCR clean-up kit (Macherey-Nagel), and sequenced using Macrogen external service. PCR primers used for amplicon generation and sequencing were designed using the Primer3 web tool [3]. Primers used for generation of the sgRNA, amplicon generation, and sequencing are listed in **Table S1**.

**Analysis of the copy number alterations and cell-type enrichment.** Copy number (CN) data was obtained from TCGA samples (BRCA-TCGA Firehose Legacy) normalized to gene level using the TCGAbiolinks (v2.28.3). Copy number alterations (CNAs) were categorized into Gain (copy number > 2), Loss (copy number < 2), or Diploid (copy number = 2). Pearson correlation between the log10(Gene expression) and the log2(CN) was performed by splitting the samples according to breast cancer subtype. Cell-type enrichment analyses, including the generation of ImmunoScore, StromaScore, and Microenvironment Score, were performed using the xCell tool [4].

**High‐throughput chromosome conformation capture (Hi-C).** Hi-C was performed following the manufacturer's protocol from Cantata Bio at the National Genomics Infrastructure, Sweden. 10^6^ cells of each cell model were fixed, digested, and cross-linked. Chromatin fragments were extracted and captured using magnetic beads to Chromatin Capture Beads. Library prep was done using the NEBNext Ultra II DNA Library Prep (Illumina). NEBNext Multiplex Oligos for Illumina were used as indexes. Sequencing was performed using NovaSeq S4, 151nt(R1)-19nt(I1)-10nt(I2)-151nt(R2). 5 kb bin contact matrices for each replicate were generated. Hi-C quality control analysis can be found here [5].

High-confidence interchromosomal interactions were defined as those interactions between Chr11q22.2 and regions located outside of chromosome 11 with an intensity higher than the first quartile of all intrachromosomal interactions between Chr11q22.2 and chromosome 11 in each sample replicates. Then, from all the potential interchromosomal interactions we selected those that overlapped with super-enhancer elements (SEE) annotated for MDA-MB-231 wild-type (Sample_02_0939) and MDA-MB-436 wild-type (Sample_02_0643) on the SEdb 2.0 database [6].

**Protein-chromatin interaction.** CTCF binding disruption was assessed by Cleavage Under Targets and Release Using Nuclease (CUT&RUN) followed by qPCR in the two cell line models [7]**.** First, cells were fixed in methanol-free 0.1% formaldehyde (Thermo Scientific) for 2 min at room temperature. Crosslinking was stopped by 1X Glycine Solution (#7005, Cell Signaling Technology (CST)) for 5 min, washed with Complete Wash Buffer (containing 1x Protease inhibitor cocktail and 1x spermidine) twice, and centrifuged at 3,000g at 4 °C for 3 min. All CUT&RUN reactions were performed using the CUT&RUN Assay Kit (#86652, CST) following the manufacturer’s instructions. Antibodies were added at the following concentrations: H3K4me3 (C42D8, CST) 1:50; CTCF (3418S, CST) 1:50; IgG isotype control (#66362, CST) 1:20. Input DNA was sonicated using 5 cycles of -15s/45s on/off- at 25% of the power. Enriched chromatin DNA samples and DNA input were purified using a DNA Purification Buffer and Spin Column system (#14209, CST) and assessed by qPCR using CFX96 Touch Real-Time PCR Detection System (Bio-Rad). H3K4me3-chromatin enrichment was used as the positive control, following the manufacturer’s guidelines. Fold enrichment was assessed by dividing CTCF-enriched into IgG-enriched chromatin extracts. PCR primers are listed in **Table S1**.

**Chromatin accessibility profiling by ATAC-seq.** 100,000 viable cells from each replicate were collected and centrifuged. Cell pellets were gently lysed for 3 min on ice. Cell nuclei were incubated for 30 min at 37ºC 1000 rpm in a thermal mixer in the presence of tagmentase. DNA products were purified using MinElut purification columns (Qiagen). Sequencing libraries were prepared following the OmniATAC protocol [8] at the National Genomics Infrastructure, Sweden. All libraries were purified using AMPure XP beads (Beckman Coulter) with a 1:1.8 ratio and sequenced in paired-end mode with a read length of 51nt(R1)-10nt(I1)-10nt(I2)-51nt(R2) on a Novaseq6000(Illumina) following the manufacturer’s instructions. Data was processed following the Galaxy project ATACseq guidelines [9]. Briefly, the raw sequencing reads were first processed using cutadapt to remove low-quality reads and adapters. Trimmed reads were then aligned to the human reference genome assembly hg38 using bowtie2. Poorly mapped reads, PCR duplicates, and reads that mapped to mitochondrial DNA were removed using BAMtools. Peaks were generated using MACS2 without building any shifting model. The resulting bedgraphs were used to generate bigwig files, which were used to visualize the peaks on the UCSC genome browser [10]. Consensus peaks for each cell line and condition were defined by their presence in all three replicates. Bedtools v2.18 was employed to determine common and exclusive peaks between each condition. Heatmaps around TSS, insulators, and enhancers were performed using the computeMatrix tool in Galaxy [11]. NCBI RefSeq genes were considered for TSS regions. Insulator elements were defined as the intersection of CTCF and RAD21 ChIP-seq peaks. The location of Enhancer elements was downloaded from the FANTOM5 website [12]. The representation factor was assessed through a hypergeometric test. The representation factor was calculated by dividing differentially accessible regions (DARs) by all the accessible regions for each genome segment (whole genome, chr11; and 20Mb, 5Mb, 1Mb around IE8). Then, all the ratios were normalized against the whole genome enrichment. The p-values were calculated using the hypergeometric test. Differences between WT and IE8dis conditions in the TSS of genes located in chr11q22.2 were represented as bigwig files loaded in the UCSC genome browser, subtracting WT signal to IE8dis in both MDA-MB-231 and MDA-MB-436. ATAC-seq quality control analysis can be found here [5].

**RNA expression analysis by RNA-seq.** Total RNA was purified using the EZNA Total RNA Kit I (Omega Biotek). For qPCR, 1 μg of RNA was retrotranscribed using SensiFAST cDNA Synthesis Kit (Bioline). qPCR reactions were performed using PerfeCTa qPCR SuperMix (Quantabio) in a CFX96 Touch Real-Time PCR Detection System (Bio-Rad). Data were normalized using GAPDH as an endogenous control. qPCR primers are listed in **Table S1**. RNA-seq was performed for three independent **replicates** of the MDA-MB-231 WT and MDA-MB-231 IE8-disrupted cell lines at NIMGENETICS, Madrid. Briefly, RNA quality was controlled by RNA ScreenTape (Agilent). All samples showed an RNA Integrity Number (RIN) equal to 10. Libraries were prepared with TruSeq stranded mRNA kit (Illumina) and sequenced in paired-end mode with a read length of 2x100bp on a HiSeq2000 (Illumina) following the manufacturer’s protocol. An average of 50 million pairs of 100-bp paired-end read per sample was generated. After adapter trimming and quality score calculation, an average of 64 to 87 million reads was left on each sample. Table counts were processed using the DEseq2 package to identify differentially expressed genes (DEGs). Gene Ontology was performed using the topGO R package. DEGs were also used to calculate the local enrichment of expression changes, comparing DEGs 1MB around IE8, the entire chromosome 11 (chr11), and the entire genome. The representation factor was calculated by dividing differentially expressed genes (DEGs) by all the genes encoded at each genome segment that were detected by RNA-seq (whole genome, chr11; and 1Mb around IE8). Then, all the ratios were normalized against the whole genome enrichment. The p-values were calculated using the hypergeometric test. RNA-seq quality control analysis can be found here [5].

**Fatty acid uptake.** The fatty acid uptake assay was performed using the QBT Fatty Acid Uptake Assay Explorer Kit (Molecular Devices), according to the manufacturer’s instructions. Briefly, 25.000 per well TNBC cells were grown in a 96-well plate at a density of 50,000 cells per well. After 24 h, cells were incubated in a serum-free medium for 1 h at 37 °C with 5% CO2. Then, 100uL QBT loading reagent was added to the plate. Plates were read using Synergy H1 microplate reader (Biotek) using bottom reading, gain 80, excitation 488, emission 528.

**MMP1 and MMP8 protein/activity level determination by ELISA.** Free Pro-MMP1 was determined using the Human Pro-MMP1 Quantikine ELISA Kit (DMP100, R&D) using Quantikine Immunoassay Control Set 656 for Human Pro-MMP-1 as an internal control (QC125, R&D). Human Total MMP-8 Quantikine ELISA Kit (DMP800B, R&D) using using Quantikine Immunoassay Control Set 968 for Human MMP-8 as an internal control (QC251, R&D). Catalytic activity of the released MMP-1 was also assessed by using Human Active MMP-1 Fluorokine E Kit (F1M00, R&D). In all the determinations, 25.000 cells were seeded in 24-well plates and cultured for 72h. After that, supernatants were collected and concentrated using speed-vacuum up to 300uL. The ELISAs were performed following the manufacturer’s guidelines. The optical density values were determined using a Synergy H1 microplate reader (Biotek). The sample signal was interpolated to a standard curve to calculate the corresponding concentration.

**Cell proliferation.** Cell viability was assessed by the Celltiter-GLO® assay (Promega). 1,000 cells/well were seeded in 96-well white opaque plates. Cell viability was assessed by adding 25uL of Celltiter reconstituted reagent on each well and measuring luminescence using microplate reader Synergy H1 (Biotek). Luminescence signals were normalized to time zero for each cell line and condition.

**Colony formation assay.** 200 cells per well were seeded in 6-well plates and incubated at 37ºC and 5% CO_2_ for 10 days. After incubation, colonies were fixed using 4% formaldehyde for 10 minutes and stained with 1% crystal violet for 1h. Wells were washed using PBS twice. After drying overnight, colonies were manually counted.

**Wound healing assay.** A longitudinal line was drawn on each well of 6-well plates before seeding 500,000 cells/well. Cells were incubated at 37ºC and 5% CO_2_. Vertical scratch wounds were performed using 200uL pipette tips once the cells reached 95% confluence. Then, wells were gently washed with PBS and continuously cultured until the wound was completely closed. Images were taken at different time points using Axio Vert.A1 inverted microscope (Zeiss). Then, images were processed using the ImageJ *Wound_healing_size_tool* plugin. Area calculations were performed following the plugin author’s guidelines [13]. Each timepoint and replicate was normalized by dividing by time zero.

**Monitor of collagen type I degradation:** The collagen type I matrix was prepared by combining 80% rat collagen I (Thermofisher), 10% PBS 10x, and 10% NaOH (0.1M). pH was adjusted to 7. Once neutralized, DQ collagen type I (Thermofisher) was added at a final concentration of 25mg/ml. Then, the collagen was mixed with a cell suspension to a final concentration of 105 cells/ml. The collagen-cell mixture was quickly dispensed into p24-well plates. All the solution reagents and materials (tips, tubes) were kept on ice. Plates were located in the incubator for 1h at 37ºC 5% CO2 to allow the collagen polymerization. Then, complete RPMI media was carefully added to the wells. After 72h, cells were fixed using paraformaldehyde 4% for 30min, carefully washed twice with PBS, and nuclei were stained with DAPI (1:10,000; Thermofisher). Images were captured using a 5x lens in the Zeiss CellObserver fluorescence microscope and analyzed using the FIJI software. First, a background subtraction was performed, and then a median filter was applied. Subsequently, the images were thresholded to obtain fluorescence regions for analysis. Images displayed were also processed with ImageJ, performing a background subtraction followed by contrast enhancement to each channel.

**Anchorage-independent growth assay.** 5,000 cells for each cell type and condition were seeded into low attachment Biofloat 96-well plates (faCellitate, Mannheim). Cells were incubated for 72h at 37ºC and 5% CO_2_ for spheroid formation. Then, cells were embedded in a mixture of cold 8mg/ml EBM (Thermofisher) and 250ug/ml of rat collagen I (Thermofisher), which was loaded into each well while the plate was placed on ice. The plate was centrifuged at 200 x g for 2min and reincubated at 37°C and 5% CO2 for one hour. Subsequently, 100uL of RPMI complete media was added to the top of the matrix mixture. Plates were incubated for 10 days. Images of spheroids were taken with an Axio Vert.A1 microscope (Zeiss) to evaluate the extent of spheroid proliferation and invasion. Spheroids were analyzed using FIJI software. Spheroid volume was calculated from the area, which was first determined by combining the threshold, magic wand, and measurement tools.

**Collagen-based cell invasion assay.** Cells were incubated for 18h in serum-free media. Cells were seeded in QCM-24-well collagen-based cell invasion assay (ECM551, Merck). The assay was performed following the manufacturer’s guidelines. Briefly, 300uL of pre-warmed serum-free media was added to the chamber inserts, which were deposited on 24-well plates. Inserts were incubated to rehydrate the collagen layer for 30 min at 37ºC and 5% CO_2_. Then, 250uL of media was replaced by 250uL of cell suspension in serum-free media containing 100,000 cells. Complete media was added to the lower chamber to act as a chemoattractant. The plate was incubated for 48h at 37ºC and 5% CO_2_ to allow cell invasion. Inserts were stained for 20 min and washed following the kit instructions. Cell staining was quantified using extraction buffer followed by optical density determination through microplate reader Synergy H1 (Biotek). Cell invasion index was normalized to MDA-MB-231 WT signal.

**hMMP1 and mutant MMP1 overexpression.** mCherry lentiviral plasmids containing either human MMP1 (hMMP1) or catalytically inactive MMP1 (mutMMP1) were sourced in VectorBuilder. Mutant MMP1 was performed by modifying four amino acids that were involved in the formation of the Zn finger close to the catalytic site (H218, E219, H222, H228). Histidine and glutamate amino acids were replaced by phenylalanine and leucine, respectively. Nucleotide substitution was performed considering codon usage frequency [14]. The selection of these amino acids was based on the literature [15,16]. hMMP1 or mutMMP1 were transfected along with psPAX2 (Addgene), and pMD2.G (Addgene) using lipofectamine 3000 (Thermofisher) following manufacturer’s instructions in HEK 293T. 72h after, lentiviral supernatant was collected, filtered, and stored until infection. 5·105 target cells were infected by spinfection (1000 rpm, 32ºC, 90 min) in presence of 1:1000 polybrene (Thermofisher). 8h later, the medium was replaced. Cells were grown during five passages. Then, mCherry cells were sorted using BD FACSAria Fusion. Functional experiments were performed as previously described except for cell proliferation, which was assessed by MTT assay. 1,000 cells/well were seeded in 96-well plates. Cell viability was assessed as follows: First, 10uL of MTT reagent were added on each well**.** Next, plates were incubated for 3h at 37ºC 5% CO_2_. After that, cell media was removed, and formazan crystals were resuspended in 100 uL of DMSO. Absorbance was determined using microplate reader Synergy H1(Biotek). Absorbance signals were normalized to time zero for each cell line and condition.

**Extended methods – References**

1. Labun K, Montague TG, Krause M, Cleuren YNT, Tjeldnes H, Valen E. CHOPCHOP v3: expanding the CRISPR web toolbox beyond genome editing. Nucleic Acids Res. 2019;47:W171–4.

2. Ran FA, Hsu PD, Wright J, Agarwala V, Scott DA, Zhang F. Genome engineering using the CRISPR-Cas9 system. Nat Protoc. 2013;8:2281–308.

3. Untergasser A, Cutcutache I, Koressaar T, Ye J, Faircloth BC, Remm M, et al. Primer3—new capabilities and interfaces. Nucleic Acids Res. 2012;40:e115–e115.

4. Aran D, Hu Z, Butte AJ. xCell: digitally portraying the tissue cellular heterogeneity landscape. Genome Biol. 2017;18:1–14.

5. Llinàs-Arias P, Ensenyat-Méndez M, Orozco JIJ, Íñiguez-Muñoz S, Valdez B, Wang C, et al. 3-D chromatin conformation, accessibility, and gene expression profiling of triple-negative breast cancer. BMC Genomic Data. 2023;24:61.

6. Wang Y, Song C, Zhao J, Zhang Y, Zhao X, Feng C, et al. SEdb 2.0: a comprehensive super-enhancer database of human and mouse. Nucleic Acids Res. 2023;51:D280–90.

7. Panday A, Elango R, Willis NA, Scully R. A modified CUT&RUN-seq technique for qPCR analysis of chromatin-protein interactions. STAR Protoc. 2022;3:101529.

8. Corces MR, Trevino AE, Hamilton EG, Greenside PG, Sinnott-Armstrong NA, Vesuna S, et al. An improved ATAC-seq protocol reduces background and enables interrogation of frozen tissues. Nat Methods [Internet]. 2017 [cited 2023 Mar 8];14:959–62. Available from: https://www.nature.com/articles/nmeth.4396

9. Delisle L, Doyle M, Heyl F. ATAC-Seq data analysis (Galaxy Training Materials) [Internet]. Galaxy Train. Netw. Galaxy Training Network; [cited 2022 Dec 16]. Available from: https://training.galaxyproject.org/training-material/topics/epigenetics/tutorials/atac-seq/tutorial.html

10. Kent WJ, Sugnet CW, Furey TS, Roskin KM, Pringle TH, Zahler AM, et al. The human genome browser at UCSC. Genome Res. 2002;12:996–1006.

11. Jalili V, Afgan E, Gu Q, Clements D, Blankenberg D, Goecks J, et al. The Galaxy platform for accessible, reproducible and collaborative biomedical analyses: 2020 update. Nucleic Acids Res. 2020;48:W395–402.

12. Lizio M, Abugessaisa I, Noguchi S, Kondo A, Hasegawa A, Hon CC, et al. Update of the FANTOM web resource: expansion to provide additional transcriptome atlases. Nucleic Acids Res. 2019;47:D752–8.

13. Suarez-Arnedo A, Figueroa FT, Clavijo C, Arbeláez P, Cruz JC, Muñoz-Camargo C. An image J plugin for the high throughput image analysis of in vitro scratch wound healing assays. PloS One. 2020;15:e0232565.

14. Nakamura Y, Gojobori T, Ikemura T. Codon usage tabulated from international DNA sequence databases: status for the year 2000. Nucleic Acids Res. 2000;28:292–292.

15. Iyer S, Visse R, Nagase H, Acharya KR. Crystal structure of an active form of human MMP-1. J Mol Biol. 2006;362:78–88.

16. Jozic D, Bourenkov G, Lim N-H, Visse R, Nagase H, Bode W, et al. X-ray structure of human proMMP-1: new insights into procollagenase activation and collagen binding. J Biol Chem. 2005;280:9578–85.
